# Whole-transcriptome insights into light-responsive non-coding RNA networks regulating circadian clock and DNA repair in zebrafish

**DOI:** 10.1007/s10142-026-01854-8

**Published:** 2026-04-25

**Authors:** Shuang Wang, Zhirui Zhu, Minjian Zou, Alessandra Boiti, Xianyong Lan, Daniela Vallone, Shengxiang Zhang, Nicholas S. Foulkes, Haiyu Zhao

**Affiliations:** 1 School of Life Sciences, Lanzhou University, No. 222 South Tianshui Road, Lanzhou, Gansu Province 730000 China; 2 Institute of Biological and Chemical Systems, Biological Information Processing (IBCS- BIP), Karlsruhe Institute of Technology (KIT), Hermann-von-Helmholtz Platz 1, Eggenstein-Leopoldshafen, 76344 Germany; 3 Key Laboratory of Animal Genetics, Breeding and Reproduction of Shaanxi Province, College of Animal Science and Technology, Northwest A&F University, No. 22 Xinong Road, Yangling, Shaanxi Province 712100 China; 4 Biocentis, Terni, Italy

**Keywords:** Light, Zebrafish model, Non-coding RNAs, Circadian clock, DNA repair, Regulatory networks

## Abstract

Light is a fundamental environmental cue that orchestrates a wide range of biological processes, from behavioral rhythms to molecular signaling pathways. Although the effects of light on entraining circadian clock and DNA repair have been well established, the post-transcriptional mechanisms involved in these responses are still not fully understood. In particular, the role of non-coding RNAs (ncRNAs), including microRNAs (miRNAs), long non-coding RNAs (lncRNAs), and circular RNAs (circRNAs), in light-regulated gene expression remains unclear. Using light-responsive whole-transcriptome RNA sequencing of zebrafish larvae, we identified 1,365 differentially expressed (DE) mRNAs, 66 DE miRNAs, 330 DE lncRNAs, and 71 DE circRNAs. RT-qPCR analyses validated the expression changes of representative transcripts. Subsequently, by integrating mRNA and ncRNA datasets, we constructed a light-responsive competing endogenous RNA (ceRNA) network, which suggested extensive miRNA-mediated interactions among coding and non-coding RNA. Additional cis- and trans-regulatory network analyses uncovered potential regulatory relationships between ncRNAs and light-responsive genes. Functional enrichment analyses of target genes highlighted key pathways, including circadian rhythm, steroid biosynthesis, phototransduction, and oxidative stress response. Subnetwork analyses further identified ncRNA-mediated regulatory networks converging on circadian clock and DNA repair. Overall, our results suggest that light exposure induces a complex post-transcriptional regulatory network in zebrafish larvae. These findings contribute to our understanding of how ncRNAs function within the circadian clock and DNA repair systems, and advance the molecular basis of light-dependent gene regulation in vertebrates.

**Supplementary Information:**

The online version contains supplementary material available at 10.1007/s10142-026-01854-8.

## Introduction

The circadian clock is an intrinsic time-keeping system that orchestrates a wide range of physiological and behavioral processes, enabling organisms to anticipate and adapt to daily environmental fluctuations imposed by the 24-hour day-night cycle (Young 2000). Light serves as the principal zeitgeber, and most plants and animals possess dedicated photoreceptive pathways that convey photic information to the molecular clockwork (Doyle and Menaker 2007; Ben-Moshe et al. 2014a). In vertebrates, the circadian clock is constructed from interlocking transcription-translation feedback loops (TTFLs) involving canonical clock genes and their protein products (Hardin et al. 1990; Sehgal et al. 1994; Aronson et al. 1994). Beyond its role in circadian clock system regulation, emerging evidence indicates that DNA repair systems are also light-sensitive and display rhythmicity under circadian control (Yang et al. 2024). Diurnal variation in DNA repair capacity has been documented across multiple organisms, suggesting a temporal optimization of genome maintenance mechanisms (Sancar et al. 2010). Light-induced changes in gene expression, particularly within central clock structures, are essential for clock entrainment, and certain light-responsive genes are co-regulated with DNA repair pathways (Hamilton et al. 2015). Although transcriptional mechanisms governing these processes have been well studied, the post-transcriptional regulation of light-induced responses, especially those involving circadian clock and DNA repair, remains to be fully defined.

Non-coding RNAs (ncRNAs), once considered transcriptional noise, are now recognized as pivotal regulators of gene expression at multiple levels (Stefani and Slack 2008). MicroRNAs (miRNAs) typically bind to the 3′ untranslated regions (3′-UTRs) of target mRNAs with partial complementarity, promoting transcript degradation or translational repression (Bartel 2009). Long non-coding RNAs (lncRNAs) and circular RNAs (circRNAs) can act as molecular “sponges”, sequestering miRNAs and thereby attenuating their repressive effects on target mRNAs through a competing endogenous RNA (ceRNA) mechanism (Salmena et al. 2011; Tay et al. 2014). LncRNAs can also modulate gene expression in *cis* or *trans* (Kopp and Mendell 2018), whereas circRNAs interact with RNA-binding proteins and can regulate their parent genes (Yang et al. 2022). Furthermore, because co-expressed genes often participate in related biological processes, the co-expression patterns between ncRNAs and mRNAs provide a critical clue to infer the regulatory roles of ncRNAs within broader functional circuits (Yuan et al. 2019). Despite increasing evidence that ncRNAs participate in clock control, the mechanisms by which light-responsive ncRNAs influence circadian clock and DNA repair remain largely unresolved.

Recent studies have underscored the regulatory potential of ncRNAs in light-dependent function and rhythmic processes. For instance, light exposure alters the expression of miR-204-3-3p and miR-430a-3p levels, which in turn modulate core clock transcripts and locomotor rhythms (Wang et al. 2025). LncRNA *HULC* perturbs the circadian oscillations by upregulating the expression of circadian oscillator CLOCK in hepatoma cells (Cui et al. 2015), and circR-*WNK2* enhances melatonin synthesis in the pineal gland by sponging miR-328a-3p and promoting *aanat* expression (Zheng et al. 2022). These examples illustrate the diversity of ncRNA-mediated control while highlighting key gaps in our understanding of their roles in light-regulated circadian clock and DNA repair systems.

The zebrafish (*Danio rerio*) represents an ideal vertebrate model for investigating photic regulation of the circadian clock and DNA repair mechanisms (Ben-Moshe et al. 2014b). Unlike mammals, zebrafish embryos, tissues, and cell lines are directly light-entrainable, allowing detailed analyses of light input pathways (Whitmore et al. 2000). The zebrafish genome encodes virtually all eukaryotic DNA repair components, including photolyases responsible for the direct reversal of UV-induced DNA damage (Pei and Strauss 2013). Importantly, both circadian clock and DNA repair pathways emerge early in development, enabling integrated functional and regulatory studies throughout embryogenesis (Ben-Moshe et al. 2014b).

In this study, we performed whole-transcriptome RNA sequencing of zebrafish larvae exposed to defined light pulses. By identifying differentially expressed mRNAs and ncRNAs, constructing light-responsive lncRNA/circRNA-miRNA-mRNA interaction and co-expression networks, and conducting functional enrichment analyses, we delineate the complex post-transcriptional regulatory landscape that links light signaling to the circadian clock and DNA repair systems. Our findings reveal new layers of light-dependent molecular regulation, offering a foundation for future mechanistic investigations into ncRNA-mediated circadian clock and genome maintenance pathways.

## Materials and methods

### Zebrafish maintenance and sample collection

Adult AB strain zebrafish (*Danio rerio*) were maintained in a recirculating water system (Thmorgan) at 28 °C under a 14 h light/10 h dark (LD) cycle. Fish were fed twice daily with freshly hatched brine shrimp. To induce spawning, we paired one male and one female fish overnight in breeding tanks with a divider, and then triggered spawning by turning on the lights the next morning. Fertilized embryos were collected, rinsed gently in E3 medium, and maintained in constant darkness (DD) until 5 days post fertilization (dpf). At 5 dpf, larvae were exposed to white light and sampled following 1, 3, or 6 h of exposure (L1H, L3H, L6H). The DD control group was sampled at 3 h, in the middle of the 6 h sampling period.

### RNA sequencing and assembly

#### RNA extraction and quality assessment

Total RNA was extracted from zebrafish larvae samples using TRIzol reagent (Invitrogen) following the manufacturer’s instructions. RNA quantity and quality were assessed using a ND-1000 spectrophotometer (NanoDrop) and an Agilent 2100 Bioanalyzer (Agilent Technologies). RNA samples with OD260/280 ≥ 1.8, OD260/230 ≥ 1.5, RNA Integrity Number (RIN) ≥ 7.0, and 28S/18S ratio ≥ 0.7 were used for library construction.

#### Library preparation and sequencing

For mRNA, lncRNA, and circRNA sequencing, 5 µg of total RNA per sample was treated with the Ribo-Zero rRNA Removal Kit (Illumina) to remove rRNAs and enrich for mRNAs and ncRNAs. The RNA was fragmented and reverse-transcribed, followed by second-strand synthesis using E. coli DNA polymerase I, RNase H, and dUTP. After A-tailing, indexed adapters with a T-overhang were ligated, and libraries were size-selected using AMPure XP beads (Beckman Coulter). PCR amplification was performed, and uracil-containing strands were digested with UDG to minimize strand bias. Libraries (300 ± 50 bp insert size) were sequenced on an Illumina Novaseq™ 6000 platform with paired-end reads. For small RNA sequencing, 5 µg of total RNA was used to construct libraries using the TruSeq Small RNA Sample Prep Kit (Illumina). Small RNAs (18–30 nt) were ligated to 3′ and 5′ adapters, reverse-transcribed, PCR-amplified, and sequenced on an Illumina HiSeq 2000/2500 platform.

#### Read mapping and transcriptome assembly

Raw reads were filtered using Cutadapt (Martin 2011) to remove adaptors and low-quality sequences. Quality was assessed using FastQC. Clean reads were aligned to the zebrafish (*Danio rerio*) reference genome (GRCz11) using Hisat2 (Kim et al. 2015) followed by transcript assembly with StringTie (Pertea et al. 2015). Small RNA-seq reads were aligned using Bowtie2 (Langmead and Salzberg 2012) for miRNA identification.

### Identification and analysis of light-responsive mRNAs

Differential expression analysis between the DD group and each light-treated group was performed on raw read counts using the *edgeR* package (version 4.1.2) (Robinson et al. 2010). Genes with an absolute log_2_ fold change (|log_2_FC|) > 0.585 (FC > 1.5 or < 0.67), and *p*-value < 0.05 were defined as significantly differentially expressed. Transcript abundance was also calculated as FPKM for visualization purposes. A heatmap depicting the expression patterns of the differentially expressed genes was generated using the gplots package (version 3.0.1). Gene Ontology (GO) and Kyoto Encyclopedia of Genes and Genomes (KEGG) enrichment analyses were performed using Metascape (www.metascape.org).

### Identification and analysis of light-responsive miRNAs

Raw miRNA reads were processed using ACGT101-miR (LC Sciences) to remove adapters, low complexity reads, and contaminants (rRNA, tRNA, snRNA, snoRNA). Filtered reads (18–26 nt) were mapped to zebrafish precursor miRNAs in miRbase 22.0 using BLAST, while unmapped reads were compared with miRNAs from other species and subsequently aligned to the zebrafish genome. Hairpin RNA structures were predicted using RNAfold. Differentially expressed miRNAs (DE miRNAs) were identified using edgeR with criteria of FC > 1.2 and *p*-value < 0.1. Targeted genes were predicted using miRanda ( < − 20 kcal/mol) and TargetScan (score percentile of > 70) (Agarwal et al. 2015; Riffo-Campos et al. 2016). Overlapping results were retained for further analysis. GO and KEGG pathway enrichment analyses of target genes were performed using Metascape.

### Identification and analysis of light-responsive lncRNAs and circRNAs

For the identification of novel lncRNAs, RNA-seq reads were first mapped to the zebrafish genome using Hisat2 (Kim et al. 2015). Subsequently, transcriptome assembly and quantification were performed using StringTie (Pertea et al. 2015). From this pool, transcripts shorter than 200 bp or those overlapping annotated protein-coding genes (mRNAs) were discarded. The coding potential of the remaining transcripts was then rigorously evaluated using CPC (Kong et al. 2007) and CNCI (Sun et al. 2013). Transcripts predicted to have coding potential by both tools (CPC score > 0 and CNCI score > 0) were removed. The final set of transcripts that passed all filters were classified as novel lncRNAs.

For the identification of circRNAs, a separate mapping strategy was employed. Clean reads were first aligned to the zebrafish reference genome using Hisat2 (Kim et al. 2015). The resulting alignment files were then independently analyzed by two established circRNA detection tools, CIRCExplorer2 (Zhang et al. 2016) and CIRI (Gao et al. 2015) which employ distinct algorithms for back-spliced junction identification. High-confidence circRNAs were defined as those consistently predicted by both tools and supported by a minimum of two unique back-spliced reads. This consensus set was subsequently merged, annotated, and used for all downstream analyses.

Differential expression of lncRNAs and circRNAs was analyzed using edgeR (FC > 1.5 or < 0.67, *p* < 0.05). Heatmaps were generated using *gplots*. miRNA-lncRNA and miRNA-circRNA interactions were predicted with miRanda and TargetScan using the same thresholds as for mRNAs. Circularity of circRNAs was validated by RNase R digestion, agarose gel electrophoresis, and RT-qPCR.

### Construction and analysis of ceRNA networks

To investigate the light-responsive post-transcriptional regulatory relationships, we constructed competing endogenous RNA (ceRNA) networks by integrating validated interactions among DE miRNAs, DE mRNAs, DE lncRNAs, and DE circRNAs. Correlation analyses among RNAs were performed to support regulatory associations. Networks were visualized using Cytoscape (v3.7.1), and functional enrichment of ceRNA-associated mRNAs was performed via Metascape.

### Co-expression and cis-regulatory networks of non-coding RNAs

Pearson correlation coefficients were calculated between DE ncRNAs (lncRNAs, circRNAs) and DE mRNAs using OmicStudio (Lyu et al. 2023). Co-expression pairs were retained when |r| > 0.9 (*p* < 0.05) for lncRNA-mRNA and |r| > 0.8 (*p* < 0.05) for circRNA-mRNA relationships. For cis-regulatory predictions, DE mRNAs located within ± 100 kbp of DE lncRNAs were considered potential targets, and pairs with |r| > 0.6 were selected. Networks were visualized with Cytoscape, and enriched GO/KEGG terms were obtained from Metascape.

### RT-qPCR validation of light-responsive non-coding RNA expression

Total RNA was extracted using TRIzol Regent (Invitrogen). Reverse transcription was performed using CoWin Biosciences kits for lncRNAs and circRNAs, and the All-in-One miRNA First Strand Synthesis Kit 2.0 (GeneCopoeia) for miRNAs. RT-qPCR was conducted using SYBR Green Master Mix (CoWin Biosciences) on a StepOnePlus Real-Time PCR System (Applied Biosystems). Primer sequences are listed in Table S1. Expression of mRNAs, lncRNAs, and circRNAs was normalized to *β-actin*, and miRNAs to *RNU6B* (*U6*). Relative expression levels were calculated using the 2^−ΔΔCt^ method (Schmittgen and Livak 2008). Each experiment was independently repeated three times.

### Statistical analysis

Statistical analyses were conducted using GraphPad Prism 8 and RStudio. Differences between two groups were assessed by Student’s *t*-test, and among multiple groups by one-way ANOVA followed by appropriate post-tests. Data are presented as mean ± SEM. A *p*-value < 0.05 was considered statistically significant. Detailed statistical information is provided in Table S2.

## Results

### Identification and functional enrichment analysis of light-responsive mRNAs

**Fig 1.**
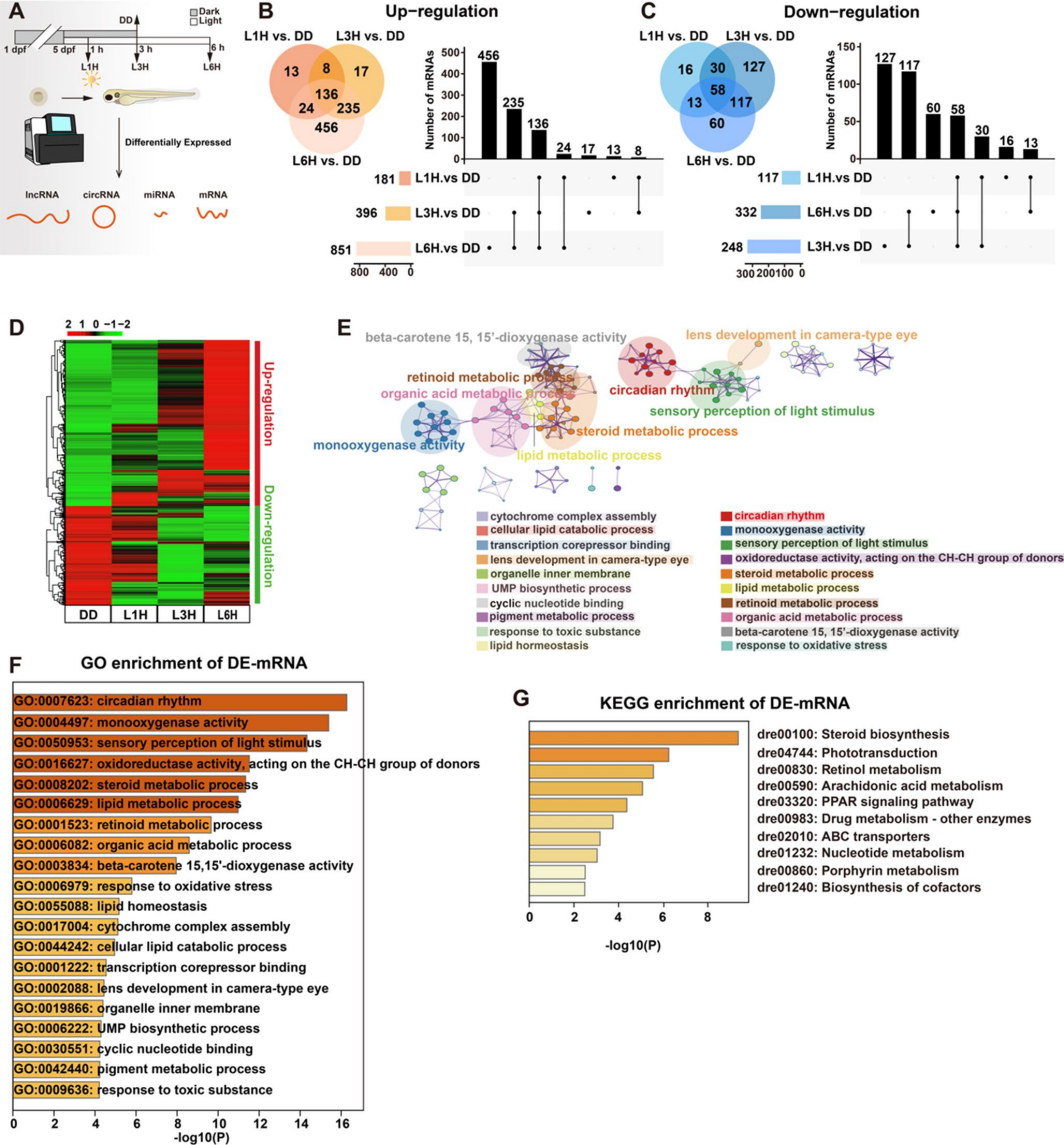
Identification and enrichment analyses of light-responsive mRNAs in zebrafish larvae.** A** Schematic of the sampling and sequencing process. **B-C** Venn diagram and UpSet plot showing up-regulated (**B**) and down-regulated (**C**) differentially expressed genes (DEGs). **D** Heatmap illustrating the expression patterns of light-responsive mRNAs. Expression levels are shown as log_2_(FPKM), with the color gradient from red to green representing high to low expression. **E-F** Gene Ontology (GO) enrichment analysis of light-responsive mRNAs. The top 20 enriched GO terms are visualized using Metascape. A representative subset of terms from the full cluster is shown as a network layout, where each circular node represents an enriched term. Node size reflects the number of associated input genes, and node color indicates cluster identity. Edges connect terms with a similarity score > 0.3. **G** KEGG pathway enrichment analysis of DE mRNAs. The top 10 enriched KEGG terms are displayed as colored bars based on *p*-values, as visualized using Metascape

To investigate the transcriptional response to light, zebrafish embryos were raised in constant darkness (DD) until 5 days post-fertilization (dpf), then exposed to light for 1 h (L1H), 3 h (L3H), or 6 h (L6H) (Fig. 1A). Whole-transcriptome sequencing was performed on 12 RNA libraries using the Novaseq™ 6000 platform. Gene expression distributions were consistent across samples, with no major deviations among cDNA libraries (Fig. S1A). The low variability in FPKM values indicated stable gene expression across replicates (Fig. S1B).

Based on the defined criteria, a total of 1,365 differentially expressed (DE) mRNAs were identified, including 889 upregulated and 476 downregulated transcripts. Venn diagrams and Upset plots illustrated group-specific and shared DE mRNAs (Fig. 1B-C). Compared to the DD group, L1H showed 181 upregulated and 117 downregulated mRNAs, L3H showed 396 upregulated and 332 downregulated mRNAs, L6H showed 851 upregulated and 248 downregulated mRNAs. Notably, the number of up-regulated genes increased with longer light exposure, with the L6H group exhibiting the largest number of upregulated genes. Further overlap analysis revealed 144 upregulated and 88 downregulated mRNAs shared between L1H and L3H, 160 upregulated and 71 downregulated mRNAs shared between L1H and L6H, 371 upregulated and 175 downregulated mRNAs shared between L3H and L6H. Across all three light treatment groups, 136 mRNAs were consistently upregulated, and 58 were commonly downregulated. A clustered heatmap illustrates the expression patterns of all significantly altered mRNAs (Fig. 1D).

To elucidate the biological functions of light-responsive mRNAs, GO and KEGG enrichment analyses were performed. GO analysis revealed significant enrichment in processes including circadian rhythm (GO:0007623), monooxygenase activity (GO:0004497), sensory perception of light stimulus (GO:0050953), steroid metabolic process (GO:0008202), and lens development in camera-type eye (GO:0002088). Network analysis highlighted overlapping biological processes such as circadian rhythm, sensory perception of light stimulus, and lens development in camera-type eye (Fig. 1E-F). KEGG pathway analysis revealed enrichment in steroid biosynthesis (dre00100), phototransduction (dre04744), and retinol metabolism (dre00830) (Fig. 1G and Fig. S1C). Together, these results demonstrate that light exposure induces specific transcriptional responses in zebrafish larvae, particularly involving genes related to circadian rhythm regulation and light transduction signaling pathways.

### Characterization of light-responsive miRNAs in zebrafish larvae

**Fig. 2:**
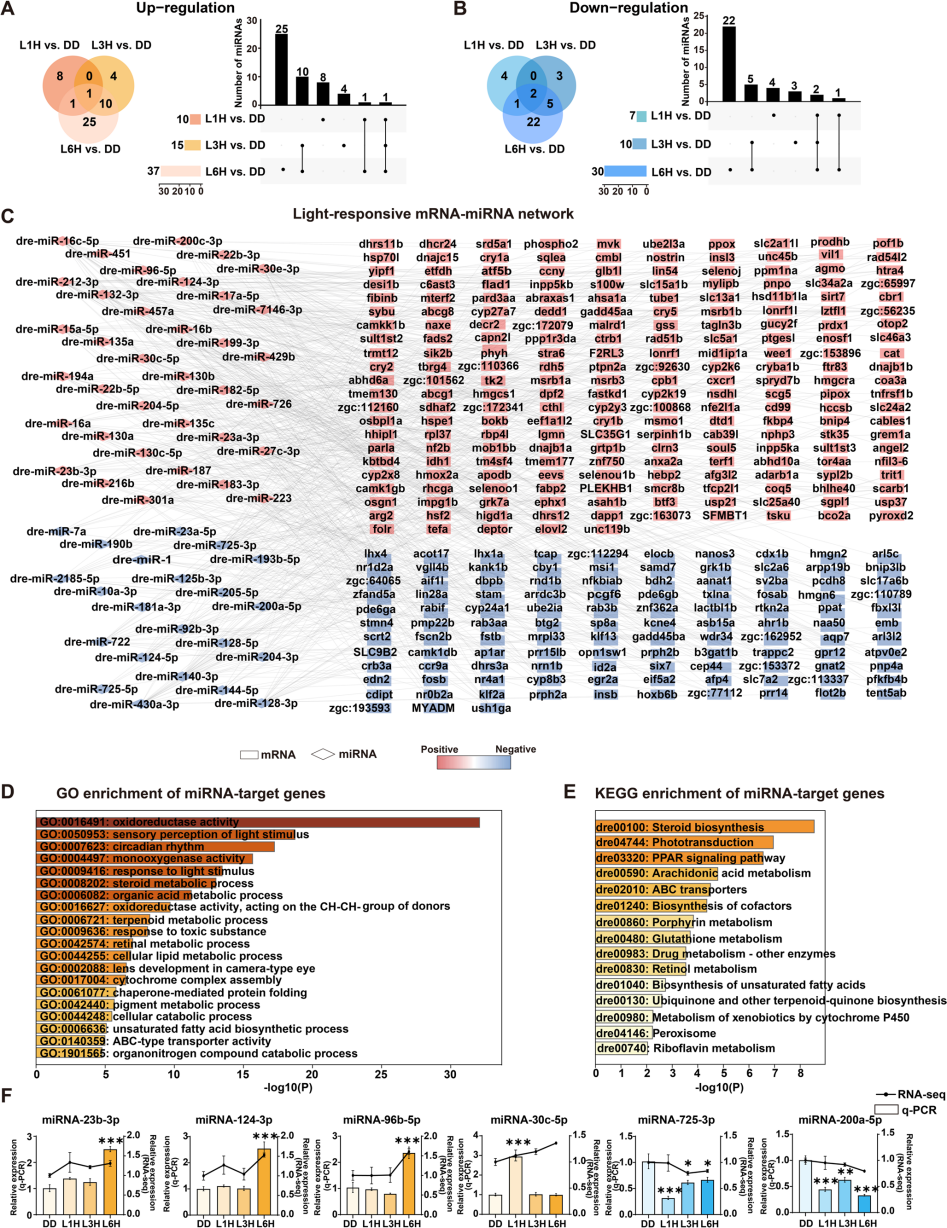
Identification and analysis of light-responsive miRNAs in zebrafish larvae.** A-B** Venn diagram and UpSet plot showing up-regulated (**A**) and down-regulated (**B**) miRNAs between L1H vs. DD, L3H vs. DD and L6H vs. DD. **C** miRNA-mRNA interaction network constructed from light-responsive miRNAs and their predicted target mRNAs. Diamonds represent miRNAs and rectangles represent mRNAs. Red nodes indicate upregulated transcripts; blue nodes indicate downregulated transcripts. **D** GO enrichment analysis of the target genes of light-responsive miRNAs, visualized using Metascape and colored by *p*-values. **E** KEGG pathway enrichment analysis of the light-responsive miRNA target genes, visualized using Metascape and colored by *p*-values. **F** RT-qPCR validation of DE miRNAs. Relative expression levels are shown as means ± SEM (*n* = 3). Each experiment was independently repeated three times. One-way ANOVA followed by Dunnett’s multiple comparisons test results are reported in Table S2. Significant differences are indicated by asterisks (* *p* < 0.05; ** *p* < 0.01; *** *p* < 0.001)

To investigate the role of miRNAs in light-induced post-transcriptional regulation, we constructed 12 small RNA libraries from four groups, each with three biological replicates. High-throughput sequencing yielded an average of approximately 12,145,021 raw reads per library. After stringent quality filtering, including the removal of low-quality reads, mRNA fragments, repeat sequences, and known non-coding RNAs from the Rfam database, an average of 5,121,591 high-quality, valid reads per library were retained for downstream analysis. A total of 1,132 miRNAs were identified across all samples. Differential expression analysis revealed a progressive increase in light-responsive miRNAs with longer light exposure durations. The most substantial changes were observed in the L6H vs. DD comparison, with 37 miRNAs upregulated and 30 downregulated. In comparison, the L3H vs. DD group exhibited 15 upregulated and 10 downregulated miRNAs, and the L1H vs. DD group showed 10 upregulated and 7 downregulated miRNAs. These results suggest a time-dependent effect of light exposure on miRNA expression in zebrafish larvae. To visualize expression overlap across conditions, Venn diagrams and UpSet plots were generated, illustrating the distribution of upregulated and downregulated miRNAs among the different light-exposed groups (Fig. 2A-B). After filtering out miRNAs with inconsistent expression patterns, a total of 66 light-responsive miRNAs were identified, among which 46 were light-induced, while 20 were repressed in response to light exposure. To explore the potential regulatory roles of these miRNAs, we predicted their target genes using miRanda and TargetScan, focusing specifically on interactions with the 3′ untranslated regions (3′-UTRs) of light-responsive mRNAs. From these predictions, 329 light-responsive mRNAs were identified as putative targets of 58 differentially expressed miRNAs. A light-responsive miRNA-mRNA interaction network was constructed using Cytoscape, revealing a complex regulatory landscape (Fig. 2C). Functional enrichment analysis of the predicted target genes showed significant enrichment in GO terms such as “oxidoreductase activity (GO:0016491)”, “sensory perception of light stimulus (GO:0050953)”, and “circadian rhythm (GO:007623)” (Fig. 2D and Fig. S2A). KEGG pathway analysis further indicated significant enrichment in “steroid biosynthesis (dre00100)”, “phototransduction (dre04744)” and “PPAR signaling pathway (dre03320)” (Fig. 2E and Fig. S2B). Additionally, six miRNAs with important regulatory roles in the network were selected for expression level verification by RT-qPCR. The results confirmed the expression trends observed in the sequencing data, supporting the reliability and reproducibility of our transcriptomic analysis (Fig. 2F). Collectively, these findings provide new insights into the miRNA-mediated post-transcriptional regulation of light-responsive processes, particularly those associated with the circadian clock, phototransduction, and metabolic pathways.

### Characterization of light-responsive lncRNAs in zebrafish larvae

**Fig. 3:**
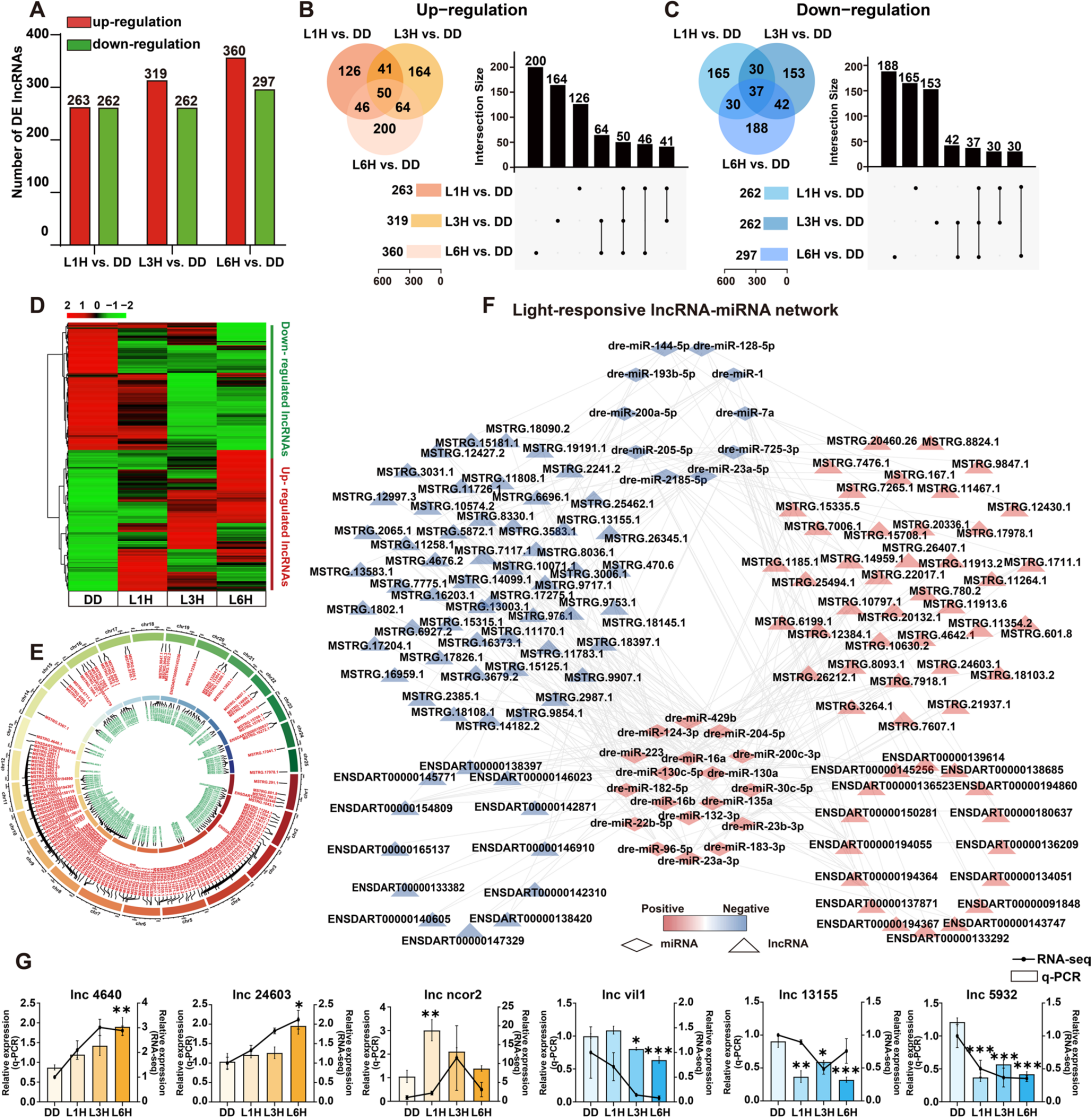
Identification and analysis of light-responsive lncRNAs in zebrafish larvae.** A** The numbers of upregulated (red) and downregulated (green) DE lncRNAs in the L1H, L3H and L6H groups compared to the DD control. **B-C** Venn diagram and UpSet plot illustrating up-regulated and down-regulated lncRNAs across different light exposure conditions. **D** Heatmap of the selected light-responsive lncRNAs. Expression levels are quantified as log_2_(FPKM). Colors range from red (high expression) to green (low expression). **E** Chromosomal distribution of light-responsive lncRNAs. **F** Predicted interaction network between light-responsive lncRNAs and miRNAs. Diamonds represents miRNAs, and triangles represent IncRNAs. Red nodes indicate up-regulated and blue nodes indicate down-regulated transcripts. **G** RT-qPCR validation of DE lncRNAs. Relative expression levels are presented as means ± SEM (*n* = 3). Each experiment was independently repeated three times. One-way ANOVA followed by Dunnett’s multiple comparisons test results are reported in Table S2. Significant differences are indicated by asterisks (* *p* < 0.05; ** *p* < 0.01; *** *p* < 0.001)

To identify long non-coding RNAs (lncRNAs), we assembled transcripts using StringTie after mapping by Hisat2 and excluded sequences shorter than 200 bp or annotated as known protein-coding mRNAs. The remaining transcripts were evaluated for coding potential using CPC and CNCI (Fig. S3A-B). Transcripts with potential protein-coding ability were excluded from further analysis as novel mRNAs. Based on these analyses, a total of 10,445 putative lncRNAs were identified, including 6,740 previously annotated and 3,705 novel lncRNAs. We then compared structural and expression characteristics between our identified mRNAs and lncRNAs. On average, lncRNAs were shorter, had fewer exons, and displayed shorter open reading frames (Fig. S3C-H), consistent with known properties of lncRNAs. To determine light-responsive lncRNAs, we performed differential expression analysis across three timepoints of light exposure (L1H, L3H, L6H) compared to DD control. In total, 1,762 differentially expressed (DE) lncRNAs were identified, with 942 upregulated and 820 downregulated. Specifically, 263 upregulated and 262 downregulated lncRNAs were identified in the L1H group, 319 upregulated and 262 downregulated in L3H, and 360 upregulated and 297 downregulated in L6H (Fig. 3A). Venn diagrams and UpSet plots were used to assess overlaps of DE lncRNAs among the light-treated groups (Fig. 3B-C). The L1H and L3H groups shared 91 upregulated and 67 downregulated lncRNAs; L1H and L6H shared 96 upregulated and 67 downregulated; L3H and L6H shared 114 upregulated and 79 downregulated DE lncRNAs. Importantly, 50 lncRNAs were consistently upregulated and 37 consistently downregulated across all three light exposure conditions. After removing lncRNAs with inconsistent or unstable expression, a final set of 330 light-responsive lncRNAs was defined, consisting of 172 upregulated and 158 downregulated transcripts. Their expression patterns across samples are visualized in a clustered heatmap (Fig. 3D, Table S3). Chromosomal mapping of these light-responsive lncRNAs revealed broad genomic distribution across all 25 zebrafish chromosomes (Fig. 3E). To explore potential regulatory roles, we constructed a lncRNA-miRNA interaction network based on predicted miRNA binding sites. The resulting network comprised 28 miRNAs and 120 lncRNAs with a total of 293 predicted interaction pairs (Fig. 3F). Finally, for lncRNA transcripts, we selected 6 representative lncRNAs, designed specific primers, and performed RT-qPCR. The results indicate that the validation outcomes are highly consistent with the high-throughput sequencing data, further confirming the reliability of the data in this study. (Fig. 3G).

### Characterization of the light-responsive circRNAs in zebrafish larvae

**Fig. 4:**
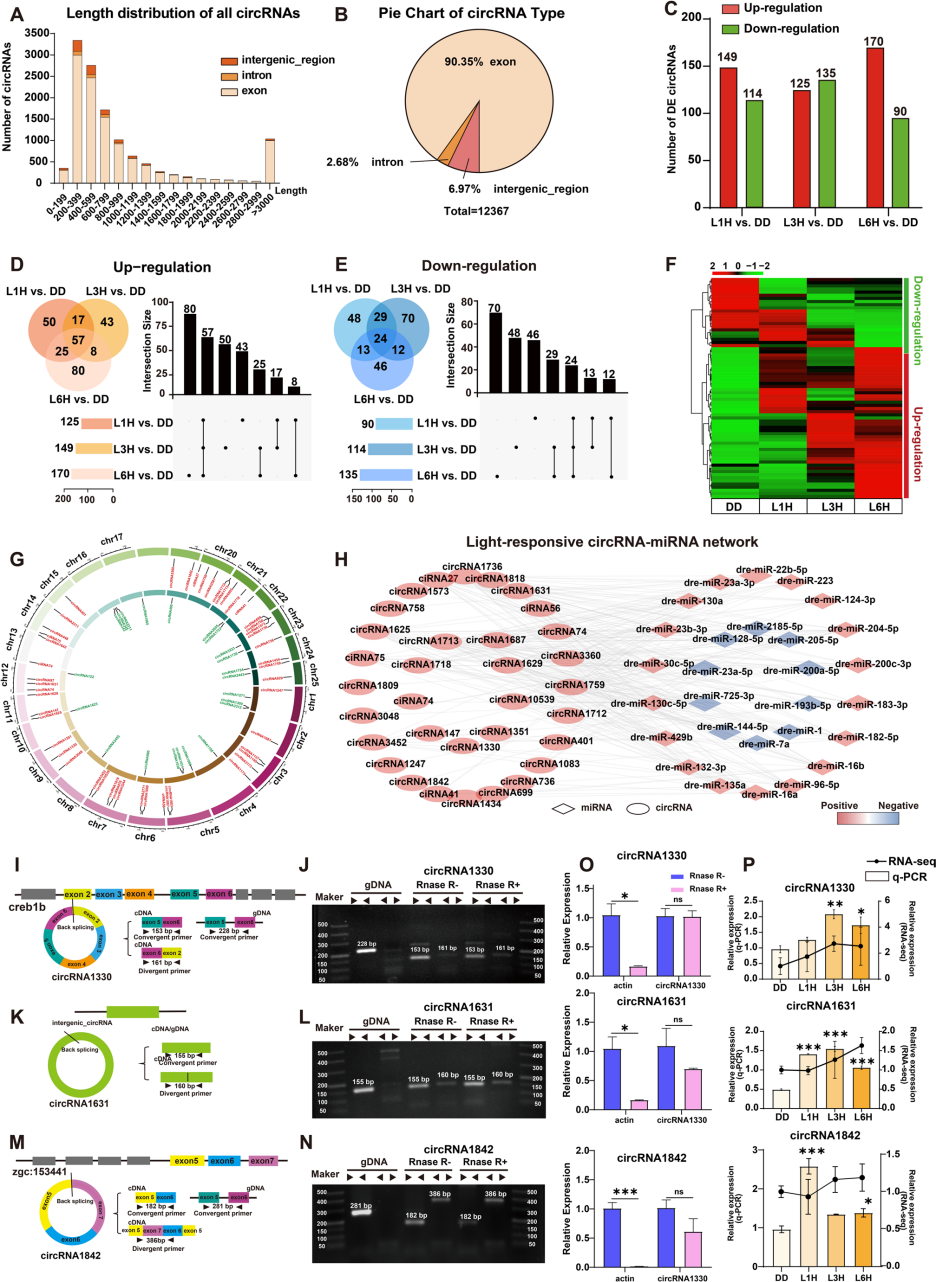
Identification and characterization of light-responsive circRNAs in zebrafish larvae.** A-B** Sequence length and genomic origin of all identified circRNAs. **C** The numbers of differentially expressed circRNAs (DE circRNAs) across comparisons. **D-E** Venn diagram and UpSet plot illustrating up- and down-regulated circRNAs. **F** Heatmap showing the expression profiles of selected light-responsive circRNAs across groups. Expression levels are quantified as log_2_(FPKM). **G** Chromosomal distribution of light-responsive circRNAs. **H** circRNA-miRNA interaction network. Diamonds represent miRNAs and ellipses represent circRNAs. **I-N** Experimental validation of circular structures. **I**, **K**, **M** Schematic diagrams of divergent and convergent primer design for circRNA1330, circRNA1631 and circRNA1842. **J**, **L**, **N** Agarose gel electrophoresis results showing amplification of circRNAs from cDNA and gDNA using divergent and convergent primers, with or without RNase R treatment. **O** RT-qPCR analysis of actin, circRNA1330, circRNA1631 and circRNA1842 before and after RNase R digestion (37 °C, 20 min) to assess RNase R resistance. **P** RT-qPCR validation of DE circRNAs. In this panel, relative circRNA expression levels are plotted on the y axes as means ± SEM (*n* = 3). Each experiment was repeated independently three times. One-way ANOVA followed by Dunnett’s multiple comparisons test results are reported in Table S2. Significant differences are indicated by asterisks (* *p* < 0.05; ** *p* < 0.01; *** *p* < 0.001)

Following the removal of low-quality, poly(N)-containing, and adaptor-contaminated reads, we obtained an average of approximately 86,028,500 clean reads per library. CircRNAs were identified using CIRCexploter2 (Ma et al. 2021) and CIRI (Gao et al. 2015), resulting in the detection of 12,367 circRNAs with lengths ranging from 42 nt to 195,759 nt (Fig. 4A). Most circRNAs (90.35%) were derived from exonic regions, while 2.68% and 6.97% originated from intronic and intergenic regions, respectively (Fig. 4B). These circRNAs were used for downstream analyses, and the DE circRNAs are listed in Table S3.

Comparison analysis between DD and the three light exposure conditions revealed 149, 125, and 170 upregulated circRNAs, and 114, 135, and 90 downregulated circRNAs in the L1H, L3H, and L6H groups, respectively (Fig. 4C). Venn and UpSet plots were used to illustrate the overlap among DE circRNAs across different treatment groups (Fig. 4D-E). Specifically, 74 upregulated and 53 downregulated circRNAs were shared between L1H and L3H, 82 upregulated and 37 downregulated circRNAs were shared between L1H and L6H, while L3H and L6H had 65 upregulated and 36 downregulated circRNAs in common. Across all three groups, 57 circRNAs were consistently upregulated and 24 were downregulated. After filtering out circRNAs with unstable expression patterns, 71 circRNAs were defined as stably light-responsive, including 49 upregulated and 22 downregulated transcripts. A heatmap visualizes their expression patterns across different groups, highlighting distinct responses to light exposure duration (Fig. 4F). Chromosomal mapping showed that light-responsive circRNAs were distributed across 24 out of 25 zebrafish chromosomes (Fig. 4G). To investigate the regulatory potential of circRNAs on miRNAs, an interaction network was constructed, revealing 283 predicted interactions among 33 circRNAs and 28 miRNAs (Fig. 4H).

Based on the back-splicing site features (Table S3), expression profiles, and functional roles in regulatory networks of circRNAs, we selected circRNA1631, circRNA1330, and circRNA1842 for experimental validation. Divergent and convergent primers targeting the back-splice junctions were designed, and their circular structures were confirmed via agarose gel electrophoresis and RNase R digestion. In cDNA, both primer types produced amplification products, whereas in genomic DNA (gDNA), only convergent primers amplified products, confirming the absence of linear transcripts. Additionally, both circRNAs were resistant to RNase R digestion, as verified by agarose gel electrophoresis (Fig. 4I-N) and RT-qPCR **(**Fig. 4O**)**, further supporting their circular nature. RT-qPCR results were consistent with the RNA-seq data, validating the expression patterns of the selected circRNAs (Fig. 4P).

### Construction of the light-responsive mRNA-miRNA-lncRNA-circRNA network

**Fig. 5:**
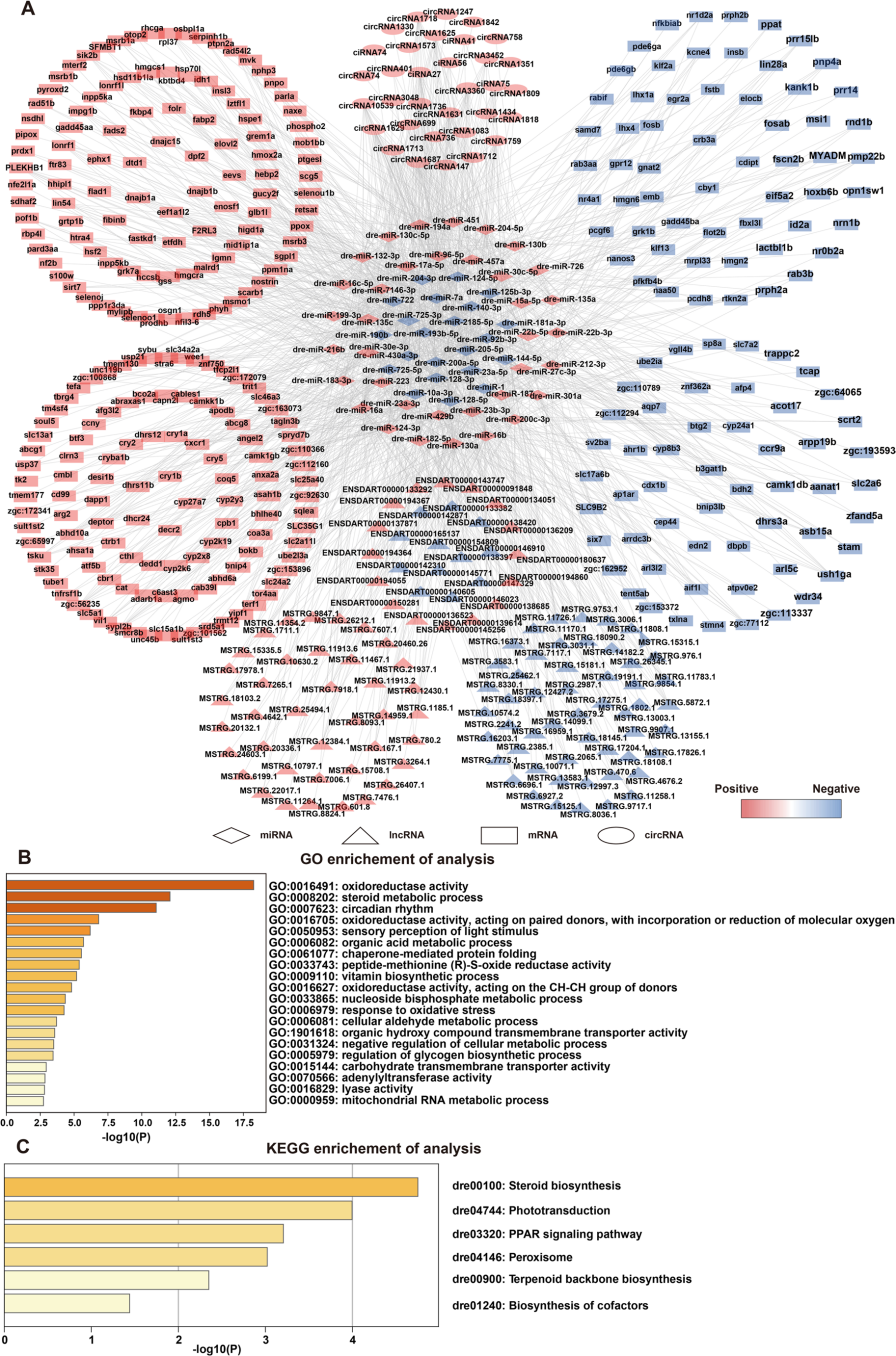
Construction and characterization of the light-responsive ceRNA network in zebrafish larvae.** A** A global view of the light-responsive ceRNA (lncRNA/circRNA-miRNA-mRNA) regulatory network. Triangles represent IncRNAs, circles represent circRNAs, diamonds represent miRNAs and rectangles represent mRNAs. Red nodes indicate up-regulated RNAs and blue nodes indicate down-regulated RNAs. **B** GO enrichment analysis of mRNAs within the ceRNA network, visualized by Metascape. Nodes are colored by *p*-values. **C** KEGG pathways enrichment analysis of mRNAs in the ceRNA network, also visualized by Metascape and colored by *p*-values

To comprehensively characterize the post-transcriptional regulatory landscape in response to light exposure, we constructed a competing endogenous RNA (ceRNA) network based on differentially expressed (DE) miRNAs, mRNAs, lncRNAs, and circRNAs. This framework leverages the ceRNA hypothesis, wherein lncRNAs and circRNAs act as molecular sponges, competitively binding miRNAs and thereby modulating the expression of their mRNA targets. Following rigorous interaction prediction and correlation filtering, we identified 329 DE mRNAs, 120 DE lncRNAs, and 33 DE circRNAs as potential targets of 58 DE miRNAs. These RNA species were then used to construct the integrated light response ceRNA network (Fig. 5A, Table S3). The resulting network highlights the intricate interplay between coding and non-coding RNAs under light exposure, revealing multiple regulatory axes and suggesting extensive cross-talk among light-responsive transcripts. GO enrichment analysis of the mRNAs targeted within the ceRNA network revealed significant enrichment of genes associated with biological processes including “oxidoreductase activity (GO:0016491)”, “steroid metabolic process (GO:0008202)” and “circadian rhythm (GO:0007623)” (Fig. 5B). Network visualization of these GO terms demonstrated functional convergence among processes such as circadian rhythm regulation, sensory perception of light stimulus, and the negative regulation of cellular metabolism, indicating shared gene modules across distinct regulatory axes (Fig. S4A). KEGG pathway analysis further revealed that ceRNA-network-associated genes were significantly enriched in pathways including “steroid biosynthesis (dre00100)”, “phototransduction (dre04744)”, and the “PPAR signaling pathway (dre03320)” (Fig. 5C). Collectively, these findings suggest that light-responsive ceRNA interactions play a likely role in coordinating key physiological processes such as circadian entrainment, metabolic adaptation and visual perception.

### Sub-networks associated with circadian clock and DNA repair regulation

**Fig. 6:**
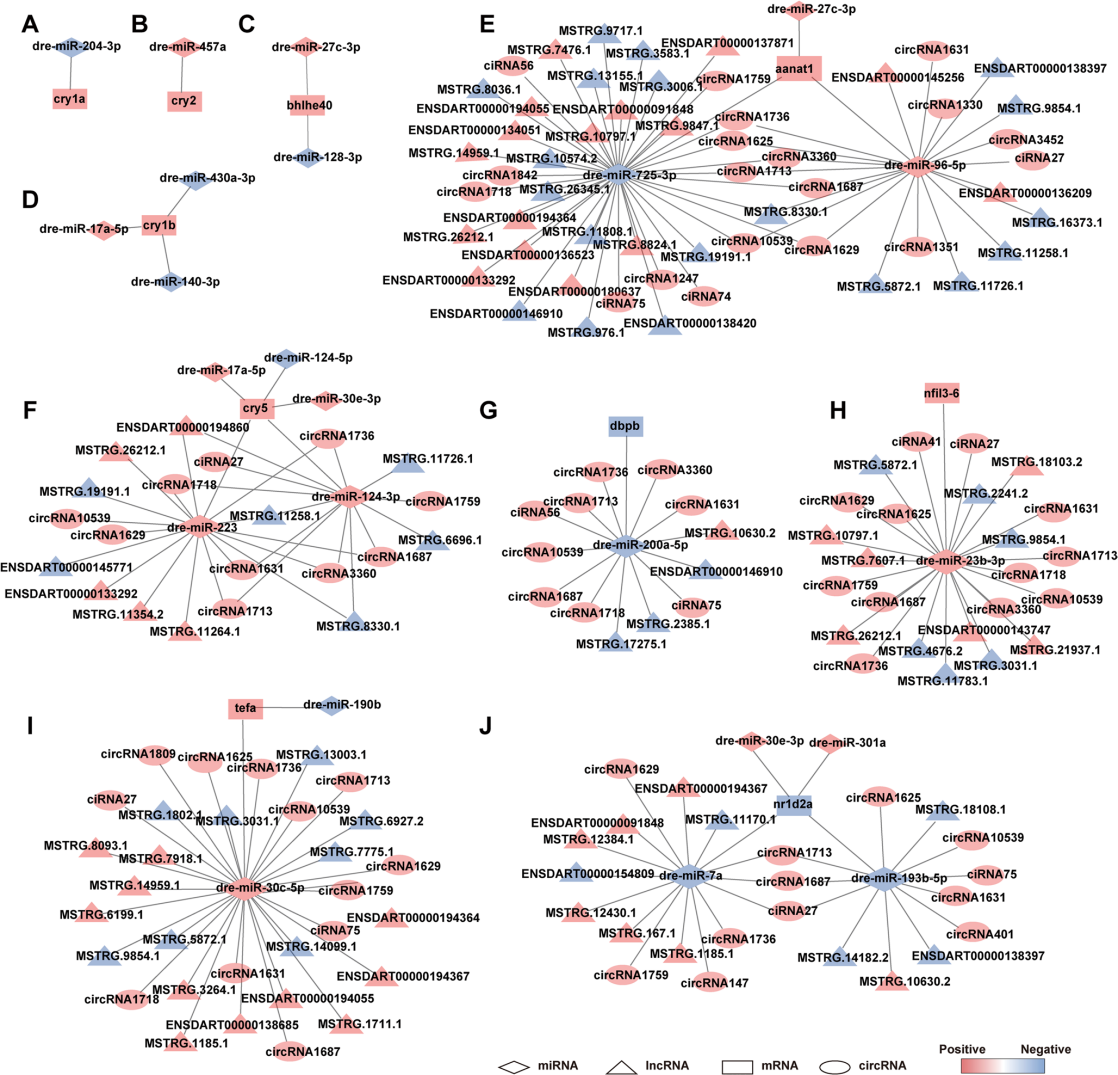
The lncRNA/circRNA-miRNA-mRNA ceRNA subnetwork associated with circadian clock and DNA repair.** A-D** Subnetworks showing miRNAs targeting key circadian clock genes. **E-J** Subnetworks showing lncRNAs and circRNAs interacting with clock and DNA repair genes through miRNA-mediated ceRNA regulation. In all panels, triangles represent lncRNAs, circles represent circRNAs, diamonds represent miRNAs, and rectangles represent mRNAs. Red nodes indicate upregulated RNAs; blue nodes indicate downregulated RNAs

To further dissect the roles of light-responsive non-coding RNAs in biological processes and regulation, we developed a series of refined ceRNA sub-networks integrating light-responsive lncRNAs, circRNAs, miRNAs, and mRNAs. Based on the ceRNA hypothesis and the identification of key hub genes, we constructed a series of core regulatory sub-networks, encompassing 69 lncRNAs, 23 circRNAs, 22 miRNAs, and 10 hub mRNAs (Fig. 6A-J). These sub-networks were specifically associated with light-regulated pathways involved in circadian rhythm and DNA repair. The hub mRNAs targeted within these networks included canonical circadian clock and photoreactivation genes including *cry1a*, *cry2*, *bhlhe40*, *cry1b*, *aanat1*, *dbpb*, *cry5*, *nfil3-6*, *tefa*, and *nr1d2a*, all of which exhibited significant differential expression following light exposure. The associated non-coding RNAs exhibited distinct expression patterns and were predicted to regulate these hub genes through miRNA-mediated interactions, forming complex post-transcriptional regulatory circuits. These light-responsive lncRNAs, circRNAs, and miRNAs may act as critical modulators in synchronizing light cues with molecular processes governing the circadian clock and genomic stability. Together, these findings outline a dynamic and multilayered non-coding RNA-mediated regulatory architecture that contributes to understanding light-induced physiological adaptation in zebrafish larvae.

### Identification and functional enrichment of lncRNA/circRNA-mRNA regulatory pairs

Given the pivotal roles of light-responsive lncRNAs and circRNAs within ceRNA core networks, we further investigated their potential regulatory relationships with mRNAs through both *cis-* and *trans-*acting mechanisms. It is well-established that genes exhibiting correlated expression profiles may share similar biological functions, a concept referred to as *trans*-regulation (Yuan et al. 2019). To explore these associations, we performed coding–non-coding co-expression network analysis by calculating Pearson correlation coefficients between differentially expressed (DE) lncRNAs/circRNAs and DE mRNAs.

**Fig. 7:**
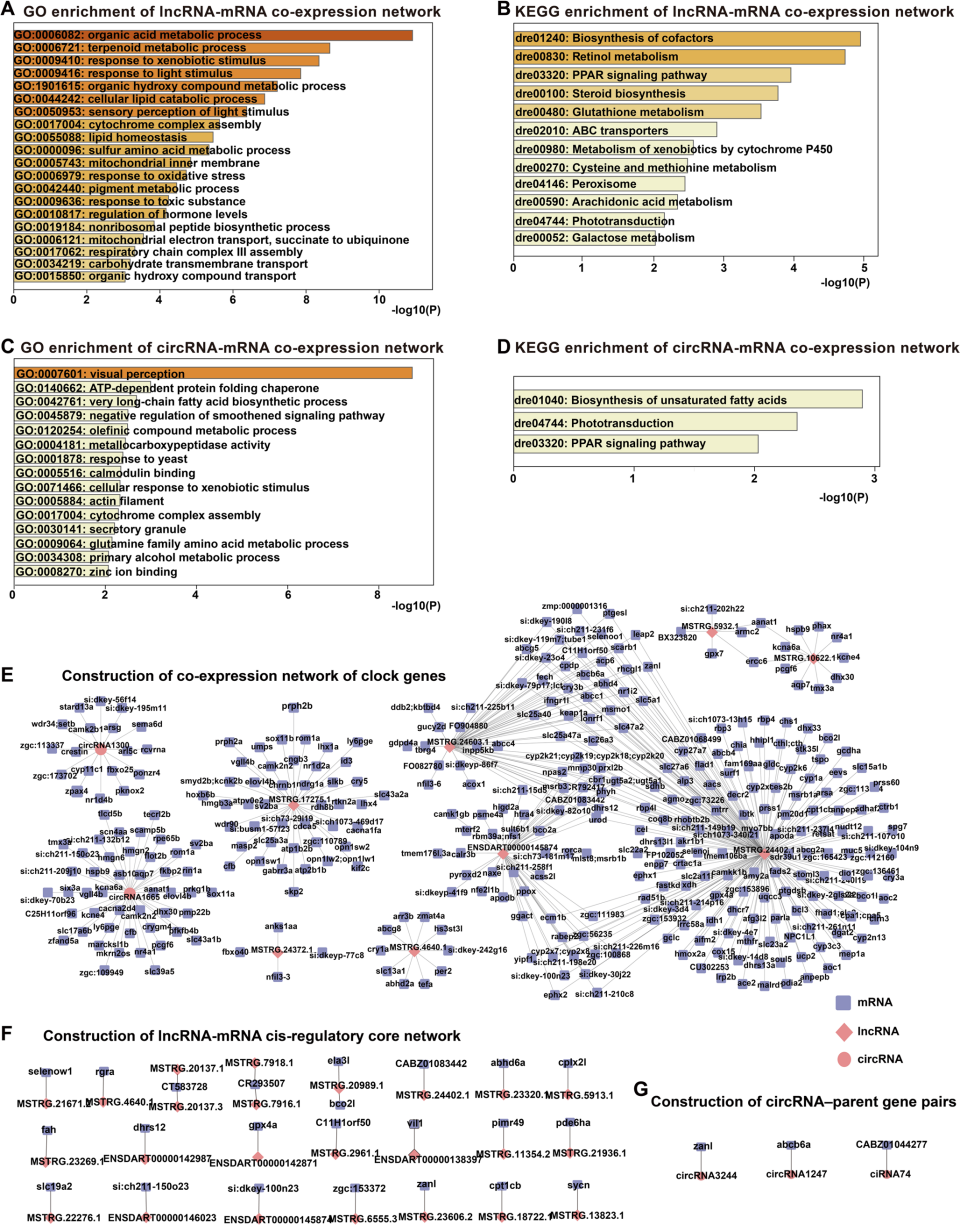
Co-expression networks and regulatory interactions between light-responsive lncRNAs/circRNAs and mRNAs in zebrafish larvae.** A** GO enrichment analysis of mRNAs co-expressed with lncRNAs, visualized by Metascape. **B** KEGG pathway enrichment analysis of co-expressed mRNAs in the lncRNA-mRNA network. **C** GO enrichment analysis of mRNAs co-expressed with circRNAs. **D** KEGG pathway enrichment analysis of mRNAs in the circRNA-mRNA network. **E** Subnetwork of lncRNA/circRNA-mRNA co-expression relationships associated with circadian clock and DNA repair genes. **F** Predicted cis-regulatory lncRNA–mRNA core network. **G** Identified circRNA-parent gene pairs, representing potential feedback regulatory interactions. In all network diagrams, triangles represent lncRNAs, circles represent circRNAs, diamonds represent miRNAs, and rectangles represent mRNAs

For lncRNAs, we identified a total of 996 significant lncRNA-mRNA co-expression pairs (|r| > 0.9, *p* < 0.05), involving 163 lncRNAs and 635 mRNAs (Fig. S5A, Table S3). GO enrichment analysis of the associated light-responsive mRNAs revealed functional enrichment in pathways such as “organic acid metabolic process (GO:0006082)”, “terpenoid metabolic process (GO:0006721)”, “response to xenobiotic stimulus (GO:0009410)” and “response to light stimulus (GO:0009416)”(Fig. 7A). KEGG pathway analysis further highlighted enrichments in “biosynthesis of cofactors (dre:01240)”, “retinol metabolism (dre:00830)” and “PPAR signaling pathway (dre:03320)” (Fig. 7B).

Similarly, co-expression analysis for circRNAs (|r| > 0.8, *p* < 0.05) identified 464 circRNA-mRNA regulatory pairs, comprising 59 circRNAs and 370 mRNAs (Fig. S5B, Table S3**)**. The target mRNAs of these circRNAs were enriched in GO terms such as “visual perception (GO:0007601)”, “ATP-dependent protein folding chaperone (GO:0140662)”, and “very long-chain fatty acid biosynthetic process (GO:0042761)” (Fig. 7C). KEGG enrichment indicated involvement in pathways including “biosynthesis of unsaturated fatty acids (dre:01040)”, “phototransduction (dre:04744)” and “PPAR signaling pathway (dre:03320)” (Fig. 7D).

To specifically examine the involvement of light-responsive ncRNAs in regulating circadian rhythm and DNA repair, we constructed a subnetwork of lncRNA/circRNA-mRNA co-expression pairs related to these two processes (Fig. 7E). This network includes regulatory relationships with core clock and DNA repair genes such as *nfil3-6*, *cry3b*, *cry1a*, *tefa*, *per2*, *aanat1*, *cry3a*, *nr1d2a*, *cry5*, *ddb2*, *xpc*, *cpdp*, *ercc6*, and *nfil3-3*. Central regulatory ncRNAs within this network were identified as potential hubs in orchestrating light-responsive gene expression dynamics.

In addition, cis-regulatory relationships were examined by identifying lncRNAs located within 100 kbp of their target genes. This analysis uncovered 22 putative *cis*-regulatory lncRNA-mRNA pairs, consisting of 24 DE lncRNAs and 23 DE mRNAs (Fig. 7F). For circRNAs, three circRNA-parent gene pairs were identified, suggesting possible feedback regulatory roles of circRNAs on their host gene expression **(**Fig. 7G**)**. Collectively, these findings offer crucial clues to the intricate interplay between ncRNAs and mRNAs in mediating the transcriptional and post-transcriptional responses to light exposure, particularly in pathways governing circadian clock biology and genome maintenance.

## Discussion

Light is a principal environmental cue that synchronizes circadian clocks with the day/night cycle (Somers et al. 1998). A central feature of this entrainment is the capacity of light to induce changes in gene expression (Tamai et al. 2007; Vatine et al. 2009). Although extensive studies have addressed the transcriptional mechanisms underlying light regulation of circadian clock and DNA repair pathways (Vallone et al. 2004; Gavriouchkina et al. 2010; Patke et al. 2020), the roles of light-responsive non-coding RNAs (ncRNAs) in these systems remain poorly understood. Previous work has revealed rhythmic expression patterns of ncRNAs in zebrafish larvae and specific organs (Ben-Moshe et al. 2014a; Mishra et al. 2021; Wu et al. 2024; Han et al. 2025), yet their mechanistic contributions to circadian regulation are largely uncharacterized. In this study, we conducted whole-transcriptome RNA sequencing and characterization in zebrafish larvae exposed to different durations of light. By integrating ceRNA, co-expression, and *cis*-regulatory networks, we identified key ncRNAs and their target genes, providing novel insights into light-driven regulatory dynamics.

### Light-responsive coding genes in circadian clock regulation

Our results demonstrated a light duration-dependent increase in the number of differentially expressed genes, supporting the sensitivity of the zebrafish transcriptome to light exposure. Compared to earlier studies using both RNA-seq and microarray platforms (Weger et al. 2011), our use of Illumina Novaseq™ 6000 enabled the detection of a broader gene set. The D-box cis-acting element has been demonstrated to mediate light-induced gene expression in zebrafish model (Weger et al. 2011; Vatine et al. 2011; Mracek et al. 2012). Here, core circadian clock and DNA repair genes, such as *cry1a*, *per2*, *ddb2* and *cry5*, known targets of D-box enhancers, were significantly upregulated upon light exposure (Drolet et al. 1991). GO enrichment highlighted “circadian rhythm” and “sensory perception of light stimulus” among significantly affected biological processes, while KEGG analysis revealed enrichment in “Phototransduction”, “Steroid biosynthesis”, and “Retinol metabolism”. These pathways are integral to light detection and circadian entrainment (Hirayama et al. 2007; Mracek et al. 2012). Many light-responsive genes are involved in overlapping functions and pathways, aligning with the evolutionary shift in vertebrates from direct light sensing by peripheral tissues to centralized, retinal-based photoreception in mammals (Menaker et al. 1978). The light-responsive modulation of retinol-related pathways reflects the importance of vitamin A derivatives in light signal transduction, corroborating their role in circadian photoreception (Besharse and McMahon 2016).

### Light-responsive non-coding RNAs integrate photic and metabolic signals into the circadian clock system

The role of non-coding RNAs in competing endogenous RNA (ceRNA) networks is a topic of growing interest (Cesana et al. 2011; Kristensen et al. 2019). In our study, we used whole transcriptome sequencing and bioinformatics tools to establish ceRNA regulatory networks, we identified 66 miRNAs, 330 lncRNAs, and 71 circRNAs, with stable light-responsive expression patterns. In the ceRNA competition mechanism, miRNA is a crucial part (Smillie et al. 2018). In our previous study, two representative light-responsive miRNAs, miR-204-3-3p and miR-430a-3p, have been demonstrated that directly regulate circadian genes. The two miRNAs can bind 3′-UTRs sites of *cry1a* and *cry1b* respectively and significantly impact the behavioral patterns and rhythmic gene expression of zebrafish larvae (Wang et al. 2025). Moreover, studies in zebrafish have shown that miR-219-5p can regulate the circadian rhythm by directly targeting *bmal1b* (Wu et al. 2024). GO analysis of predicted miRNA target genes revealed significant enrichment in “oxidoreductase activity”, suggesting that light-induced oxidative stress might be a key factor driving post-transcriptional regulation. These miRNAs appear to participate in adaptive responses to light-induced oxidative stress, potentially contributing to the modulation of the circadian clock system. Additionally, many terms related to metabolic pathways were present in the enrichment results. These findings suggest that light-responsive miRNAs play a crucial role in integrating metabolic and oxidative stress pathways into the regulation of circadian clock system. According to the ceRNA hypothesis, circRNA and lncRNA can serve as ceRNA to compete for miRNA response elements, thus regulating the expression level of target genes. Compared with the enrichment analysis of light-responsive mRNAs, the ceRNA network was further concentrated on pathways related to oxidative stress, metabolic homeostasis, and the circadian clock, including “oxidoreductase activity”, “steroid metabolic process”, and “circadian rhythm”. Notably, the PPAR signaling pathway was significantly enriched in the KEGG analysis. The PPAR pathway is closely associated with lipid metabolism and oxidative stress. In many organisms, the PPAR signaling pathway can be activated by external stimuli and subsequently participates in the regulation of clock genes (Jordan et al. 2017; Wang et al. 2022). These results suggest that, through non-coding RNAs, light may dynamically coordinate the interactions among metabolic homeostasis, oxidative stress, and the circadian clock system within the ceRNA regulatory network.

In addition, we investigated the potential biological functions of light-responsive non-coding RNAs by constructing lncRNA-mRNA and circRNA-mRNA co-expression networks and performing functional enrichment analysis on their target genes. The results revealed that the lncRNA-mRNA interaction network was significantly enriched in processes such as “sensory perception of light stimulus” and “retinol metabolism”. Notably, the circRNA-mRNA network was significantly enriched in “visual perception” and “phototransduction”, underscoring the broad involvement of ncRNAs in light response mechanisms. As a key environmental zeitgeber, light perception and transduction are the first steps of circadian entrainment. The perception and transduction of light signals represent the starting point for organisms to regulate circadian rhythms (Yoshii et al. 2015; De Magalhaes Filho et al. 2018). Our findings imply that both lncRNAs and circRNAs are embedded within the light-responsive regulatory network, contributing to visual perception, phototransduction, and circadian rhythm synchronization. Future work should employ functional experiments to validate the specific mechanisms of key circRNAs/lncRNAs in phototransduction and circadian oscillation, providing new insights into the network functions of non-coding RNAs in light-regulated physiology.

### Non-coding RNA-mediated regulation of the circadian clock and DNA repair

Network analysis suggested that light-responsive non-coding RNAs regulate core clock genes, including *cry1a*/*b*, *cry2*, *clock2 cry3a*/*b*, *per2*, *aanat1*, *dbpb*, *nfil3-3*/*6*, *tefa*, and *nr1d2a*. The *cry* and *per* family members function as central repressors within the zebrafish TTFL, whereas genes like *dbpb*, *nfil3-3*, *nfil3-6*, *tefa*, and *nr1d2a* serve as critical components in the circadian auxiliary loops (Vatine et al. 2011). In the subnetwork analysis, miR-725-3p, miR-27c-3p, and miR-96-5p were identified as potential direct regulators of *arylalkylamine N-acetyltransferase 1* (*aanat1*). *Aanat* and its encoded protein AANAT are involved in melatonin synthesis. Melatonin synthesis is under circadian control, and melatonin itself functions as an important chronobiotic that plays a crucial role in regulating the daily activity patterns of organisms (Seth and Maitra 2010). miR-96-5p belongs to the miR-183/96/182 cluster, among which miR-183 has been demonstrated to target the 3′-UTR of the pineal clock-controlled gene *aanat2* and regulate its rhythmic mRNA expression (Ben-Moshe et al. 2014a). Our study suggests that light-responsive lncRNAs and circRNAs may indirectly regulate *aanat*. Overall, light regulates circadian rhythm through multiple ncRNA-mediated pathways. Notably, our study reveals that the role of light-responsive ncRNAs extends beyond the circadian clock to include the important biological process of DNA repair. Through further analysis of the ceRNA and co-expression networks, we found that light-responsive non-coding RNAs would potentially regulate a series of key DNA repair genes, including *cry5*, *cpdp*, *ddb2*, *xpc*, and *ercc6*. Among them, *cry5* encodes a 6-4 photolyase whose expression is light-induced, consistent with its role in light-dependent DNA repair (Sancar 2008), and a recent study also confirmed the dual regulatory function of *cry5* in​ circadian clock and DNA repair systems (Li et al. 2025b). In our study, multiple non-coding RNAs were predicted to interact with *cry5*. Furthermore, the CPD photolyase encoded by the *cpdp* has been demonstrated to mediate the photoactivated repair of UV-induced DNA lesions and also performs the light-independent repair of oxidative stress-induced DNA damage (Li et al. 2025a). Moreover, DNA repair genes such as *ddb2*, *xpc*, and *ercc6*, which are involved in nucleotide excision repair (NER) and base excision repair (BER) pathways (Zhao et al. 2018, 2021), were also identified, suggesting broader regulatory connections between light-responsive non-coding RNAs and DNA repair mechanisms. These results indicate that light-responsive ncRNAs could directly or indirectly mediate post-transcriptional regulation within the circadian clock system, while also exerting potential influence on DNA repair system.

It should be noted that the ceRNA/co-expression networks constructed in this study are based on a limited sample size. This may lead to some instability in the correlation estimates within the networks and pose a risk to the inferred regulatory relationships. Therefore, we emphasize that the network model derived from this study should be regarded as an exploratory analysis and hypothesis-generating tool, rather than a definitive confirmation of regulatory interactions. Its core value lies in screening high-priority candidate regulatory axes from a transcriptome-wide perspective, thereby providing clear targets and direction for subsequent direct validation through functional experiments. Future studies should employ CRISPR/Cas9-based loss- or gain-of-function approaches in zebrafish models to validate the regulatory roles of key miRNAs, lncRNAs, and circRNAs on circadian clock and DNA repair genes. Moreover, in vivo imaging and tissue-specific expression profiling could further elucidate the spatiotemporal dynamics of these ncRNA-mediated regulatory networks.

## Conclusions

**Fig. 8:**
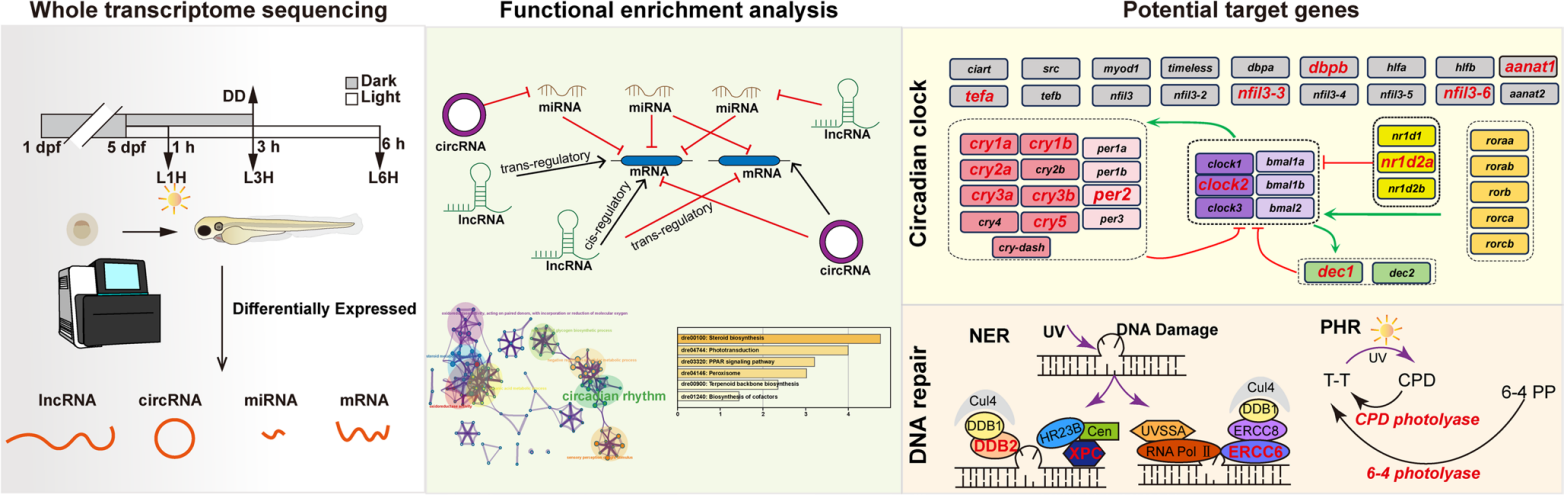
This diagram illustrates the potential mechanisms of light-responsive non-coding RNAs in the regulation of the circadian clock and DNA repair systems

Zebrafish serves as an ideal vertebrate model for exploring light-regulated biological processes. In this study, we generated expression profiles of light-responsive coding and non-coding RNAs in zebrafish larvae through whole-transcriptome RNA sequencing. By integrating differentially expressed mRNAs, miRNAs, lncRNAs, and circRNAs, we constructed light-responsive ceRNA and co-expression networks. We specifically focused on sub-networks associated with the circadian clock and DNA repair systems. This study highlights the complex interplay between light-responsive non-coding RNAs, and advances our understanding of how non-coding transcriptomes contribute to environmental adaptability via circadian clock and genome maintenance (Fig. 8). Although we have validated the expression and presence of selected non-coding RNAs, the network model presented in this study remains preliminary predicted. Its primary value lies in prioritizing hypotheses for subsequent functional experiments.

## Supplementary Information

Supplementary Material 1

Supplementary Material 2

## Data Availability

The datasets supporting the conclusions of this article are included within the manuscript and its additional files. The whole-transcriptome RNA sequencing raw data have been submitted to the National Center for Biotechnology Information (NCBI). The raw RNA-seq data (PRJNA1120586), previously analyzed for mRNA expression, were reanalyzed here for circRNA and lncRNA discovery. The miRNA sequencing data are available under accession number PRJNA1120592.

## Abbreviations

ncRNA: Non-coding RNA
SCN: Suprachiasmatic nucleus
*per*: *Period*
*cry*: *Cryptochrome*
mRNA: Messenger RNA
miRNA: MicroRNA
lncRNA: Long non-coding RNA
circRNA: Circular RNA
ceRNAs: Competing endogenous RNAs
DD: Constant Darkness
DE mRNA: Differentially Expressed mRNA
DE miRNA: Differentially Expressed miRNA
DE lncRNA: Differentially Expressed lncRNA
DE circRNA: Differentially Expressed circRNA
FC: Fold Change
GO: Gene Ontology
ROS: Reactive Oxygen Species
TTFL: Transcription-Translation Feedback Loop
*aanat*: *Arylalkylamine N-acetyltransferase*
RT-qPCR: Real‑Time Polymerase Chain Reaction
U6: RNU6B
