# Supplementary material for: Whole-transcriptome insights into light-responsive non-coding RNA networks regulating circadian clock and DNA repair in zebrafish
Source: Funct Integr Genomics. 2026 Apr 25;26(1):86. doi: 10.1007/s10142-026-01854-8 (PMC13110244; doi:10.1007/s10142-026-01854-8)
Supplement: Supplementary file 1 — Supplementary Material 1 [file 10142_2026_1854_MOESM1_ESM.zip › Additional file 1.docx]

**Whole-Transcriptome Insights into Light-Responsive Non-Coding RNA Networks Regulating Circadian Clock and DNA Repair in Zebrafish**

Shuang Wang^1,4^, Zhirui Zhu^1,4^, Minjian Zou^1^, Alessandra Boiti^2#^, Xianyong Lan^3^, Daniela Vallone^2^, Shengxiang Zhang^1^, Nicholas S. Foulkes^2,^*****, Haiyu Zhao^1,^*****

**^1^** School of Life Sciences, Lanzhou University, No. 222 South Tianshui Road, Lanzhou 730000, Gansu Province, China.

**^2^** Institute of Biological and Chemical Systems, Biological Information Processing (IBCS-BIP), Karlsruhe Institute of Technology (KIT), Hermann-von-Helmholtz Platz 1, 76344 Eggenstein-Leopoldshafen, Germany.

**^3^** Key Laboratory of Animal Genetics, Breeding and Reproduction of Shaanxi Province, College of Animal Science and Technology, Northwest A&F University, No. 22 Xinong Road, Yangling 712100, Shaanxi Province, China.

**^4^** These authors contributed equally to this work.

# Current address: Biocentis, Terni, IT

*** Corresponding authors:**

Haiyu Zhao Email: [zhaohy@lzu.edu.cn](mailto:zhaohy@lzu.edu.cn)

Nicholas S. Foulkes Email: [nicholas.foulkes@kit.edu](mailto:nicholas.foulkes@kit.edu)

**Table S1.** Sequences of primers used in this study.

|  | **Gene name** | **Sequences of the primers (5’ – 3’)** | **Product length** |
| --- | --- | --- | --- |
| miRNA | U6 | R: CTCGCTTCGGCAGCAGCACA | 20 |
|  | miRNA23b-3p | R: GCATCACATTGCCAGGGAT | 19 |
|  | miRNA-124-3p | R: TAAGGCACGCGGTGAATG | 18 |
|  | miRNA-96-5p | R: GGCGGTTTGGCACTAGCAC | 19 |
|  | miRNA-30c-5p | R: GCAGTGTAAACATCCTACACTCT | 23 |
|  | miRNA-725-3p | R: CGCAGTTCAGTCATTGTTTC | 20 |
|  | miRNA-200a-5p | R: CATCTTACCGGACAGTGCT | 19 |
| lncRNA | lnc 24603 | F: TGAACACACGCATGCAATGG | 156 |
|  |  | R: AAGCCACCGTTTGCATTGTC |  |
|  | lnc 4640 | F: CACTGTTGTGCATCATGGCTA | 176 |
|  |  | R: TGACACTTTCTGTGGTTTCGT |  |
|  | lnc ncor2 | F: CAGTATGGGATCTCGTTCTCCG | 148 |
|  |  | R: AGGTTGGCTCTGGTTGAACTC |  |
|  | lnc vil1 | F: ATAGGAAGGAAGTTCCAGGGAAG | 130 |
|  |  | R: GGAGTTCAGAGAAGATGAGCGTG |  |
|  | lnc 13155 | F: CGTCCACTTCCATTAGCCGT | 195 |
|  |  | R: CCTTGTGAATGTGCAGCGTC |  |
|  | lnc 5932 | F: GTGTGTCCGTTTATGTGGTGTC | 165 |
|  |  | R: CTGTCTGATGCCCTGTAGGAAG |  |
| circRNA | circRNA1330-convergent primer | F: CTCGCAGGAGTCAGTAGACAG | 153 |
|  |  | R: GCAGTGTCCTCCTCAGATTTC |  |
|  | circRNA1330-divergent primer | F: TCTTGAACGACCTGTCATCAG | 161 |
|  |  | R: CTTTACTCCTCTGCTACTGCG |  |
|  | circRNA1631- convergent primer | F: CTCATAGGTCGGAGGATGACG | 155 |
|  |  | R: CTGCATCTGTTTCCACGCAAC |  |
|  | circRNA1631- divergent primer | F: ATGCAGAAGAATATTGCGGACA | 160 |
|  |  | R: TTACAACAGCCCCAAATCAAGT |  |
|  | circRNA1842- convergent primer | F: GCTGTTCACTCGAGAGTTAGC | 182 |
|  |  | R: TCTGAGCACCTTGATAGGGTG |  |
|  | circRNA1842- divergent primer | F: GCTGTTCACTCGAGAGTTAGC | 386 |
|  |  | R: CGGTAGCTCACCAATGAATCG |  |

**Table S2.** Detailed statistical analysis in this study.

| **The expression of miRNAs (Figure. 2F)** | | | | | |
| --- | --- | --- | --- | --- | --- |
| miRNA vs. U6 | | one-way ANOVA |  | |  |
| miRNA-23b-3p | DD | Dunnett’s multiple comparisons | F=39.17; P<0.0001 *** | | N=3 |
|  | DD vs. L1H | - | P=0.0909 | ns | N=3 |
|  | DD vs. L3H | - | P=0.3312 | ns | N=3 |
|  | DD vs. L6H | - | P<0.0001 | *** | N=3 |
| miRNA-124-3p | DD | Dunnett’s multiple comparisons | F=29.35; P<0.0001 *** | | N=3 |
|  | DD vs. L1H | - | P=0.9034 | ns | N=3 |
|  | DD vs. L3H | - | P=0.9996 | ns | N=3 |
|  | DD vs. L6H | - | P=0.0001 | *** | N=3 |
| miRNA-96-5p | DD | Dunnett’s multiple comparisons | F=22.78; P=0.0003 *** | | N=3 |
|  | DD vs. L1H | - | P=0.9773 | ns | N=3 |
|  | DD vs. L3H | - | P=0.5649 | ns | N=3 |
|  | DD vs. L6H | - | P=0.0007 | *** | N=3 |
| miRNA-30c-5p | DD | Dunnett’s multiple comparisons | F=180.0; P<0.0001 *** | | N=3 |
|  | DD vs. L1H | - | P<0.0001 | *** | N=3 |
|  | DD vs. L3H | - | P=0.9952 | ns | N=3 |
|  | DD vs. L6H | - | P=0.9996 | ns | N=3 |
| miRNA-725-3p | DD | Dunnett’s multiple comparisons | F=13.27; P=0.0018 ** | | N=3 |
|  | DD vs. L1H | - | P=0.0006 | *** | N=3 |
|  | DD vs. L3H | - | P=0.0169 | * | N=3 |
|  | DD vs. L6H | - | P=0.0326 | * | N=3 |
| miRNA-200a-5p | DD | Dunnett’s multiple comparisons | F=32.84; P<0.0001 *** | | N=3 |
|  | DD vs. L1H | - | P=0.0002 | *** | N=3 |
|  | DD vs. L3H | - | P=0.0022 | ** | N=3 |
|  | DD vs. L6H | - | P<0.0001 | *** | N=3 |
|  | | | | | |
| **The expression of lncRNAs (Figure. 3G)** | | | | | |
| lncRNA vs. *β-actin* | | one-way ANOVA |  | |  |
| lnc 24603 | DD | Dunnett’s multiple comparisons | F=4.992; P=0.0368 * | | N=3 |
|  | DD vs. L1H | - | P=0.8759 | ns | N=3 |
|  | DD vs. L3H | - | P=0.7219 | ns | N=3 |
|  | DD vs. L6H | - | P=0.0213 | * | N=3 |
| lnc 4640 | DD | Dunnett’s multiple comparisons | F=5.904; P=0.020 * | | N=3 |
|  | DD vs. L1H | - | P=0.4628 | ns | N=3 |
|  | DD vs. L3H | - | P=0.1378 | ns | N=3 |
|  | DD vs. L6H | - | P=0.0086 | ** | N=3 |
| lnc ncor2 | DD | Dunnett’s multiple comparisons | F=9.852; P=0.0046 ** | | N=3 |
|  | DD vs. L1H | - | P=0.0027 | ** | N=3 |
|  | DD vs. L3H | - | P=0.0638 | ns | N=3 |
|  | DD vs. L6H | - | P=0.7276 | ns | N=3 |
| lnc vil1 | DD | Dunnett’s multiple comparisons | F=21.94; P=0.0003 *** | | N=3 |
|  | DD vs. L1H | - | P=0.3850 | ns | N=3 |
|  | DD vs. L3H | - | P=0.0322 | * | N=3 |
|  | DD vs. L6H | - | P=0.0009 | *** | N=3 |
| lnc 13155 | DD | Dunnett’s multiple comparisons | F=15.32; P=0.0011 ** | | N=3 |
|  | DD vs. L1H | - | P=0.0014 | ** | N=3 |
|  | DD vs. L3H | - | P=0.0294 | * | N=3 |
|  | DD vs. L6H | - | P=0.0008 | *** | N=3 |
| lnc 5932 | DD | Dunnett’s multiple comparisons | F=46.83; P<0.0001 *** | | N=3 |
|  | DD vs. L1H | - | P<0.0001 | *** | N=3 |
|  | DD vs. L3H | - | P=0.0001 | *** | N=3 |
|  | DD vs. L6H | - | P<0.0001 | *** | N=3 |
|  | | | | | |
|  | | | | | |
| **The expression of circRNAs (Figure. 4O)** | | | | | |
| R+ vs. R- | | Unpaired t test |  | |  |
| circRNA1330 | actin | R+ vs. R- | P=0.0137 | * | N=3 |
|  | circRNA1330 | R+ vs. R- | P=0.9649 | ns | N=3 |
| circRNA1631 | actin | R+ vs. R- | P=0.0137 | * | N=3 |
|  | circRNA1631 | R+ vs. R- | P=0.2735 | ns | N=3 |
| circRNA1842 | actin | R+ vs. R- | P=0.0007 | *** | N=3 |
|  | circRNA1842 | R+ vs. R- | P=0.2125 | ns | N=3 |
|  | | | | | |
| **The expression of circRNAs (Figure. 4P)** | | | | | |
| circRNA vs. *β-actin* | | one-way ANOVA |  | |  |
| circRNA1631 | DD | Dunnett’s multiple comparisons | F=137.8; P<0.0001 *** | | N=3 |
|  | DD vs. L1H | - | P<0.0001 | *** | N=3 |
|  | DD vs. L3H | - | P<0.0001 | *** | N=3 |
|  | DD vs. L6H | - | P<0.0001 | *** | N=3 |
| circRNA1330 | DD | Dunnett’s multiple comparisons | F=8.985; P=0.0061 ** | | N=3 |
|  | DD vs. L1H | - | P=0.4779 | ns | N=3 |
|  | DD vs. L3H | - | P=0.0036 | ** | N=3 |
|  | DD vs. L6H | - | P=0.0277 | * | N=3 |
| circRNA1842 | DD | Dunnett’s multiple comparisons | F=36.51; P<0.0001 **** | | N=3 |
|  | DD vs. L1H | - | P<0.0001 | *** | N=3 |
|  | DD vs. L3H | - | P=0.1021 | ns | N=3 |
|  | DD vs. L6H | - | P=0.0273 | * | N=3 |
|  | | | | | |


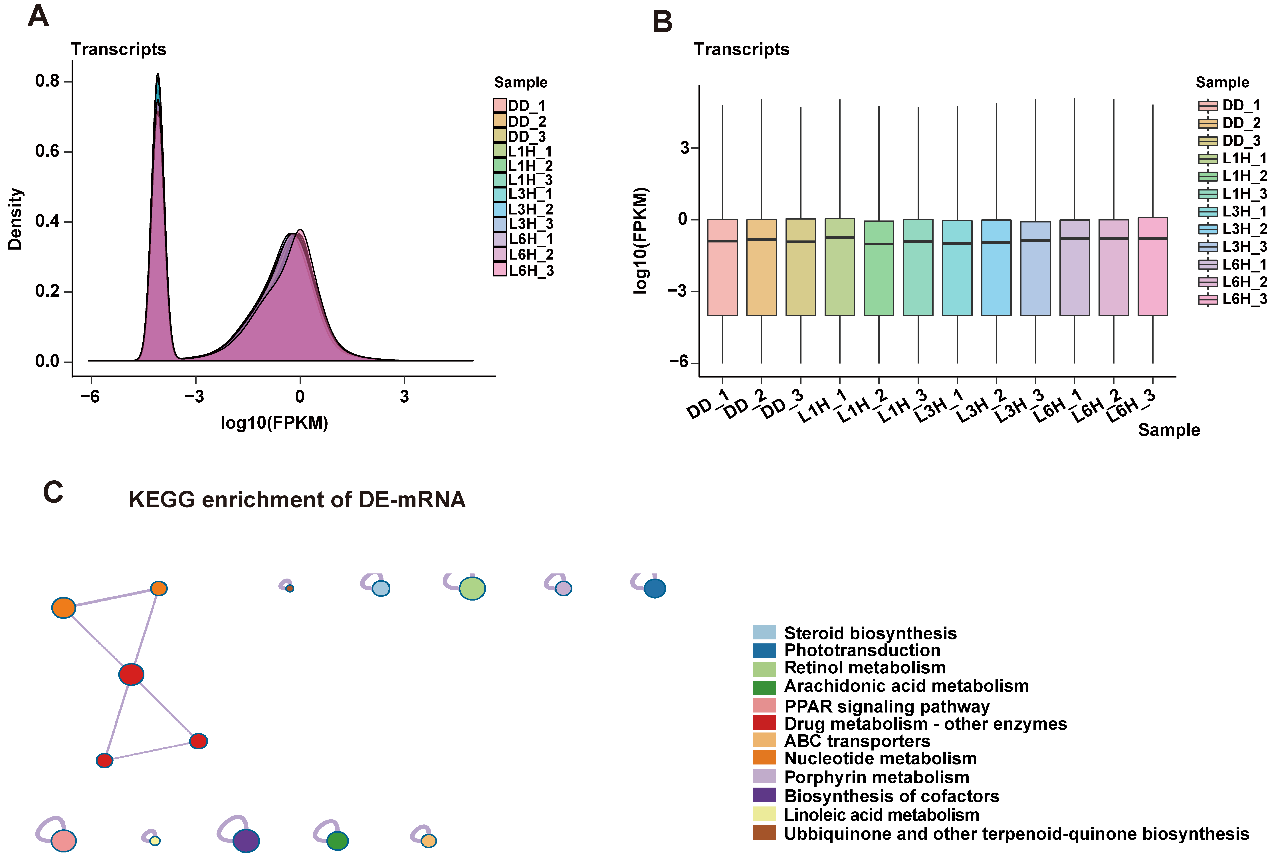


**Fig.S1**: **A** FPKM density distribution of the sequencing data. The x-axis represents the log10 (FPKM) value of the gene, and the y-axis represents the distribution density of the genes with corresponding expressions. **B** Boxplot of the FPKM density distribution. **C** The top 10 KEGG pathways enriched terms of the DE mRNAs. A representative subset of terms is selected from the entire cluster and converted into a network layout. Each term is represented by a circular node whose size is proportional to the number of input genes under that term, and whose color indicates its cluster identity. Terms with a similarity score > 0.3 are connected by an edge.


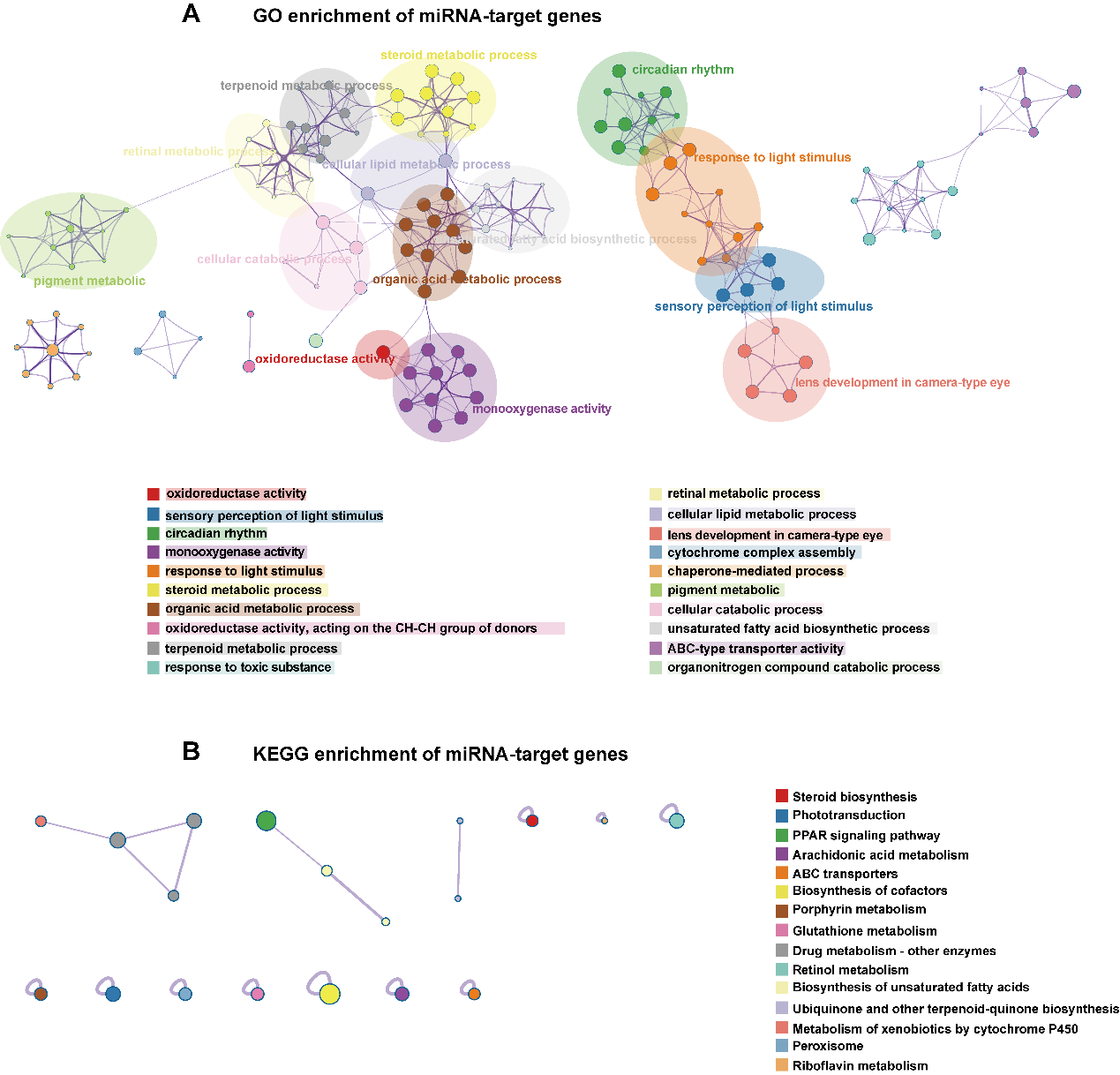


**Fig.S2 A** The Gene Ontology (GO) enriched terms of the miRNA-target genes. **B** The KEGG pathways enriched terms of the miRNA-target genes.


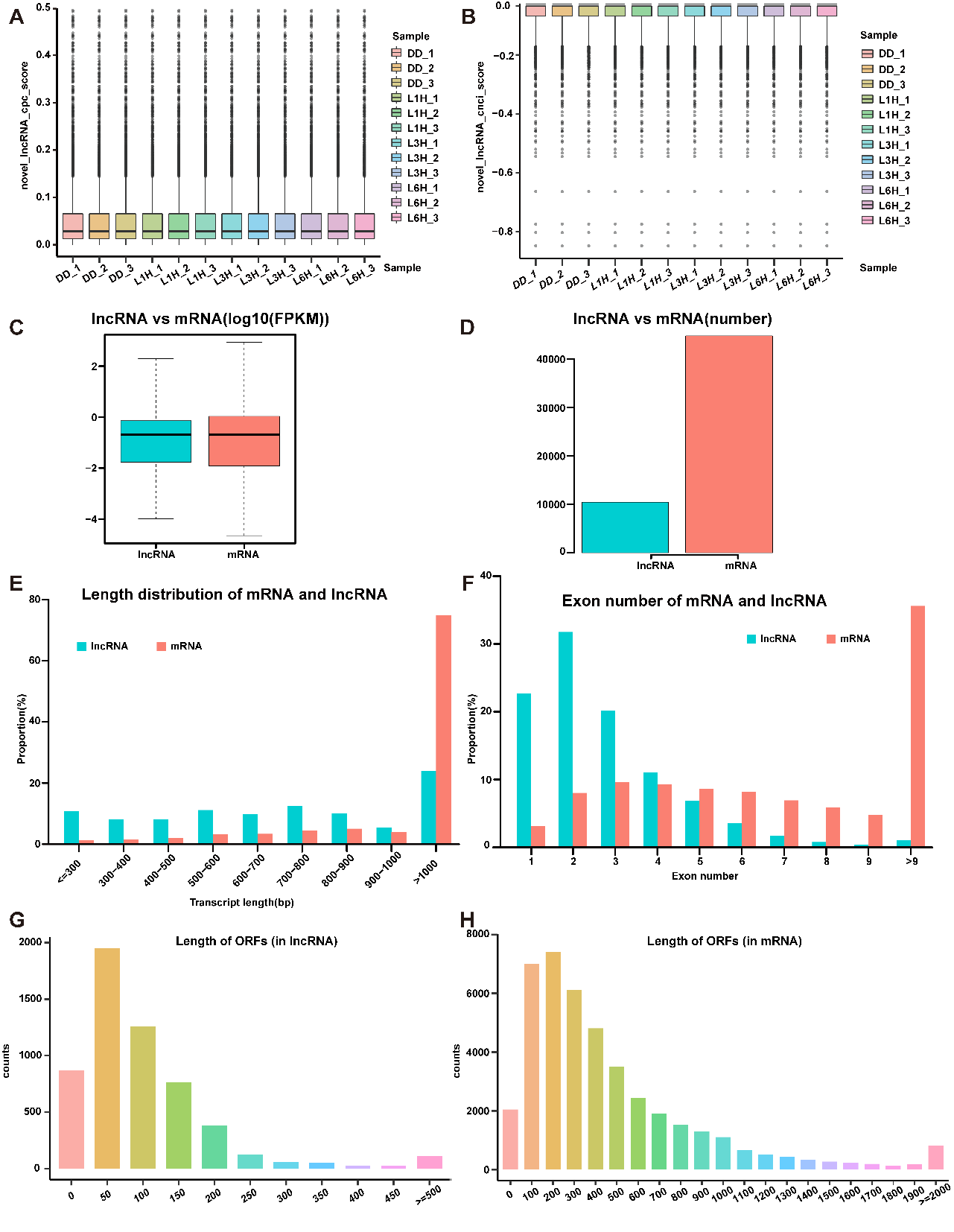


**Fig. S3**: **A-B** novel lncRNA CPC (**A**) score boxplot and CNCI (**B**) score boxplot.

**C** The expression level of all mRNA and lncRNA**. D** The number of all mRNA and lncRNA.

**E** Length distribution of all mRNA and lncRNA. **F** Exon number of all mRNAs and lncRNAs.

**G-H** Open reading frame (ORF) length in lncRNA (**G**) and mRNA (**H**).


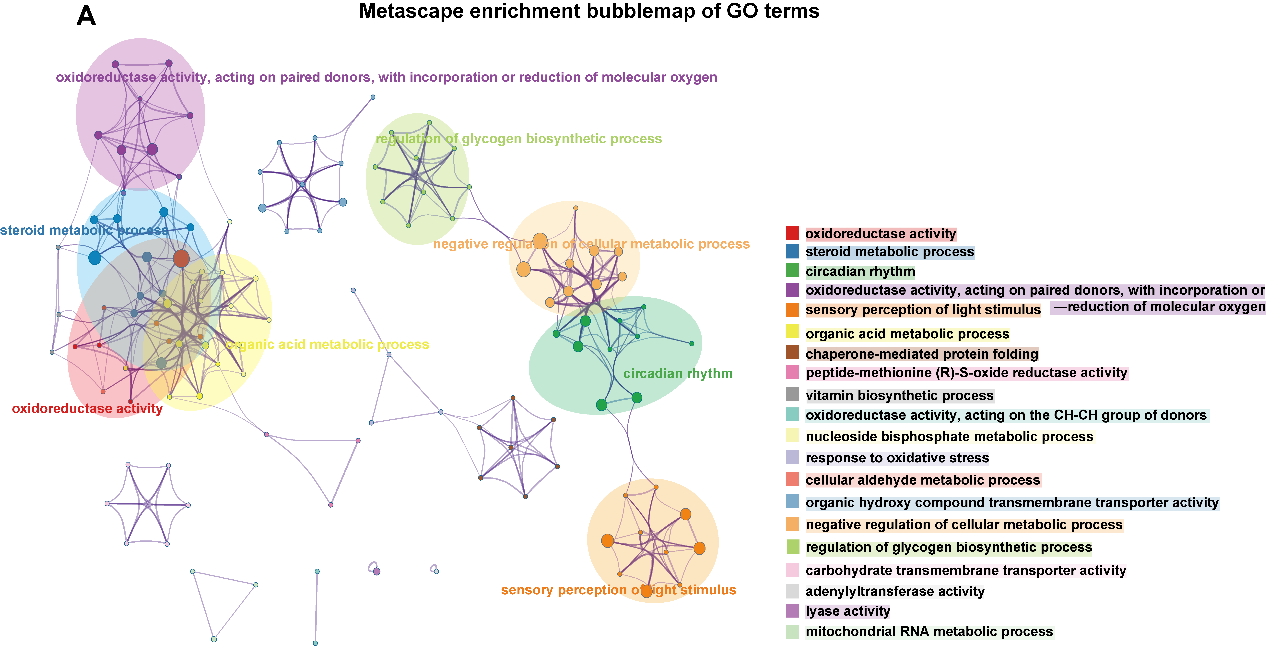


**Fig. S4：The GO enriched terms and KEGG enrichment analysis of target mRNAs in the ceRNA network.** **A** The Gene Ontology (GO) enriched terms of the ceRNA network by Metascape, colored by p-values. A representative subset of terms is selected from the entire cluster and converted into a network layout. Each term is represented by a circular node whose size is proportional to the number of input genes under that term, and whose color indicates its cluster identity. Terms with a similarity score > 0.3 are connected by an edge.


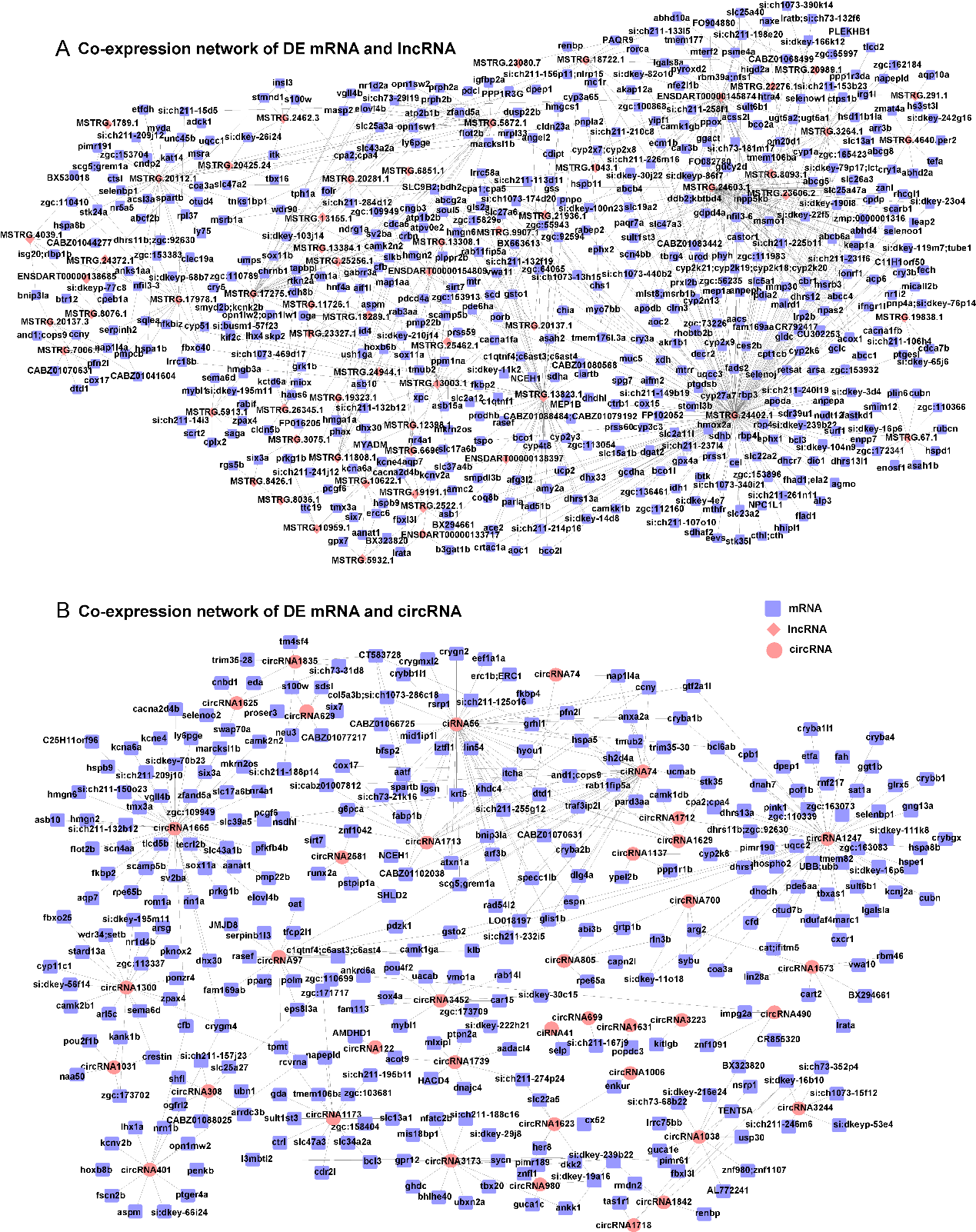


**Fig.S5：Co-expression network of lncRNAs/circRNAs-mRNA. A** Co-expression network of lncRNAs-mRNAs. **B** Co-expression network of circRNAs-mRNAs.
